# CircLRFN5 inhibits the progression of glioblastoma via PRRX2/GCH1 mediated ferroptosis

**DOI:** 10.1186/s13046-022-02518-8

**Published:** 2022-10-20

**Authors:** Yang Jiang, Junshuang Zhao, Rongqing Li, Yingliang Liu, Lin Zhou, Chengbin Wang, Caihong Lv, Liang Gao, Daming Cui

**Affiliations:** 1 Department of Neurosurgery, Shanghai Tenth People’s Hospital, Tongji University School of Medicine, Shanghai, 200072 China; 2 Department of Neurosurgery, Taihe Affiliated Hospital of Hubei University of Medicine, Shiyan, 442000 China

**Keywords:** GSCs, Ferroptosis, CircLRFN5, PRRX2, GCH1

## Abstract

**Background:**

Ferroptosis is a novel form of iron-dependent cell death and participates in the malignant progression of glioblastoma (GBM). Although circular RNAs (circRNAs) are found to play key roles in ferroptosis via several mechanisms, including regulating iron metabolism, glutathione metabolism, lipid peroxidation and mitochondrial-related proteins, there are many novel circRNAs regulating ferroptosis need to be found, and they may become a new molecular treatment target in GBM.

**Methods:**

The expression levels of circLRFN5, PRRX2 and GCH1 were detected by qPCR, western blotting, and immunohistochemistry. Lentiviral-based infections were used to overexpress or knockdown these molecules in glioma stem cells (GSCs). The biological functions of these molecules on GSCs were detected by MTS (3-(4, 5-dimethylthiazol-2-yl)-5-(3-carboxymethoxyphenyl)-2-(4-sulfophenyl)-2H tetrazolium), the 5-ethynyl-20-deoxyuridine (EdU) incorporation assay, transwell, neurosphere formation assays, Extreme Limiting Dilution Analysis (ELDA) and xenograft experiments. The content of ferroptosis levels in GSCs was detected by BODIPY 581/591 C11 assay, glutathione (GSH) assay and malondialdehyde (MDA) assay. The regulating mechanisms among these molecules were studied by RNA immunoprecipitation assay, RNA pull-down assay, ubiquitination assay, dual-luciferase reporter assay and chromatin immunoprecipitation assay.

**Results:**

We found a novel circRNA circLRFN5 is downregulated in GBM and associated with GBM patients’ poor prognosis. CircLRFN5 overexpression inhibits the cell viabilities, proliferation, neurospheres formation, stemness and tumorigenesis of GSCs via inducing ferroptosis. Mechanistically, circLRFN5 binds to PRRX2 protein and promotes its degradation via a ubiquitin-mediated proteasomal pathway. PRRX2 can transcriptionally upregulate GCH1 expression in GSCs, which is a ferroptosis suppressor via generating the antioxidant tetrahydrobiopterin (BH4).

**Conclusions:**

Our study found circLRFN5 as a tumor-suppressive circRNA and identified its role in the progression of ferroptosis and GBM. CircLRFN5 can be used as a potential GBM biomarker and become a target for molecular therapies or ferroptosis-dependent therapy in GBM.

**Supplementary Information:**

The online version contains supplementary material available at 10.1186/s13046-022-02518-8.

## Introduction

Glioblastoma (GBM) is the most malignant tumor in the central nervous system, with a WHO grade of IV in all gliomas [1]. Although surgery combined with radiotherapy and chemotherapy is the most common therapy in GBM, the median survival time is still less than 15 months, which seriously threatens patients’ health and life safety [2]. Glioma stem cells (GSCs) are a kind of tumor-initiating cells with multipotent, self-renewal and expressed with stem cell markers, such as CD133 and nestin [3]. GSCs play an essential role in the occurrence, development, recurrence and chemoradiotherapy tolerance of GBM [4].

Circular RNAs (circRNAs) are a particular class of non-coding RNAs (ncRNAs), which form a circular structure by covalent bonds without a 5’ end cap and 3’ end poly (A) tail in linear RNAs [5]. CircRNAs are characterized by high stability, abundance, and conservation in all kinds of human tissues, especially in brains [6]. Abnormal expression of circRNAs is found in glioma, gastric cancer, pancreatic cancer and other tumors, and they are reported to play a vital role in the progression, metastasis and other malignant phenotypes of tumors [7–9]. For example, circNHSL1 promotes gastric cancer progression through the miR-1306-3p/SIX1/vimentin axis [8]. CircPDK1 promotes pancreatic cancer glycolysis via c-myc activation by modulating miR-628-3p/BPTF axis and degrading BIN1 [9]. Our previous study found circARF1 was overexpressed in glioma and regulated its angiogenesis [10]. CircATP5B can regulate the proliferation of GSCs via the IL6-mediated JAK2/STAT3 signaling pathway [11]. However, compared with the enormous numbers of circRNAs, only a few circRNAs with definite functions have been studied, and there are still many circRNAs with unknown functions to be further studied in GBM or GSCs.

Ferroptosis is a novel form of iron-dependent regulated cell death (RCD) and is utterly different from apoptosis, necrosis, autophagy, and other forms of cell death [12]. Ferroptosis is characterized by the accumulation of intracellular lipid-reactive oxygen species (ROS) [13]. The polyunsaturated fatty acids (PUFAs) in mitochondria, endoplasmic reticulum and lysosomes can be oxidized by lipoxygenase and ROS to form lipid hydroperoxide (L-OOH) [14]. In the presence of iron, the L-OOH turns into toxic lipid free radicals (L-O-), leading to the fragmentation of PUFAs in the membrane lipids and cell death [14]. Iron metabolic imbalance and ferroptosis are significantly associated with the malignant progression of various tumors. For example, ferroptosis inducer erastin enhances the sensitivity of GBM to temozolomide [15]. The regulation of ferroptosis in tumors may become a new method for tumor therapy, but the detailed mechanisms need to be further clarified. In glioma, circCDK14 can inhibit ferroptosis and promotes its progression via regulating PDGFRA [16]. In other types of tumors, circPVT1 knockdown increases ferroptosis and promotes 5-Fluorouracil chemosensitivity via miR-30a-5p/FZD3 axis in esophageal cancer cells [17]. CircLMO1 suppresses cervical cancer growth and promotes cervical cancer cell ferroptosis through up-regulating ACSL4 expression [18]. However, as master regulators of various cellular processes [19], it is worth studying novel circRNAs regulate ferroptosis in GSCs.

Paired related homeobox 2 (PRRX2) is a transcription factor (TF) of the paired family of homeobox proteins. PRRX2 was firstly found to be expressed in proliferating fetal fibroblasts and the developing dermal layer, with a role in fetal skin development and a possible role in cellular proliferation [20]. PRRX2 was also reported to promote colon cancer liver metastasis via activation of Wnt/β-catenin signaling [21]. PRRX2 expression was upregulated in prostate cancer and acted as a regulator of enzalutamide resistance [22]. However, there was no study about PRRX2 expression and function in GBM.

In our study, we firstly found a novel circRNA, hsa_circ_0031751 (named circLRFN5), was significantly downregulated in GBM tissues and negatively correlated with GBM patients' poor prognosis. The aim of our study was to investigate the role and demonstrate the potential mechanism of circLRFN5 in GSCs. CircLRFN5 may be used as a potential GBM biomarker and a treatment target for molecular and ferroptosis-dependent therapy. We also provided mechanistic insights into the regulation between circRNAs and ferroptosis.

## Materials and methods

### Patient samples and ethical approval

Seventy glioma tissues were obtained from patients diagnosed with glioma and who underwent surgery in the Department of Neurosurgery of Shanghai Tenth People’s Hospital. Among them, according to the World Health Organization (WHO) classification guidelines, 20 samples of grade II, 25 samples of grade III, and 25 samples of grade IV glioma. Moreover, ten adjacent normal brain tissues (NBT) of GBM patients were also collected as normal control. All participants provided written informed consent, and the research was approved by the Ethics Committee of Shanghai Tenth People’s Hospital.

### Cell culture

Six patient-derived primary GSCs from WHO grade IV specimens (GSC51, GSC52, GSC53, GSC55, GSC56 and GSC58) were isolated and validated as previously described [23]. The detailed clinical information for these samples is outlined in Table S1. Briefly, freshly resected GBM tissues were dissociated into single cells using type IV collagenase. Then the dissociated cells were maintained in serum-free DMEM/F12 with 2% B27, 20 ng/mL rh-bFGF, and rh-EGF (Gibco, Gaithersburg, MD, USA) for two weeks. After tumorspheres formation, the stem cell markers and multi-lineage differentiation capacities were detected to validate the characteristics of GSCs. To avoid any inferences, all GSCs have passed mycoplasma and the short tandem repeat (STR) DNA profiling test. GSCs in all assays were cultured in less than 20 generations. The GSCs were treated with several reagents, including necrostatin-1 (an inhibitor of necroptosis, 20 μM), Z-VAD-FMK (a pan-caspase inhibitor, 20 μM), 3-Methyladenine (3-MA, an inhibitor of autophagy, 60 μM), ferrostatin-1 (Fer-1, a ferroptosis inhibitor, 10 μM), and RSL (a ferroptosis activator, 2 μM). All these reagents were purchased from MedChemExpress (MCE, Monmouth Junction, NJ, USA).

### Lentiviral vector construction and transfection

The overexpression of circLRFN5, PRRX2, and GCH1 was constructed using the lentivirus-based vectors by Gene-Chem (Shanghai, China). The silence of circLRFN5 and PRRX2 were constructed using RNAi-mediated lentivirus vectors (Gene-Chem). After transfection, all GSCs were selected at a concentration of 10 μg/ml puromycin (Sigma, Santa Clara, CA, USA) for 15 days. The lentivirus transfection efficacy was validated by qPCR or western blotting [23]. The sequences of all siRNAs are listed in Table S2.

### qRT-PCR (real-time quantitative reverse transcription PCR)

As previously described [23], the total RNAs of GSCs were extracted using a Mini-BEST Universal RNA Extraction kit (TaKaRa, Kyoto, Japan). The amount and quality of isolated RNAs were detected by Nanodrop (Thermo Fisher Scientific). The cDNA library was constructed using Prime-Script RT Master Mix Kit (TaKaRa). The qRT-PCR was performed using a SYBR Green Master Mix Kit (TaKaRa) via PCR LightCycler480 (Roche Diagnostics, Basel, Switzerland). The β-actin was used as an endogenous control. Primers used in this study are listed in Table S3.

### RNase R assay

RNase R assay was performed as previously described [23]. Briefly, 2 μg total RNA was incubated with 5U/μg RNase R (Epicentre Technologies, Madison, WI, USA) at 37 °C for 30 min. Then the expression of circRNAs and their linear form RNAs were detected by qPCR.

### Western blotting

Western blotting was performed as previously described [23]. First, the total protein of GSCs was isolated using the total cell protein extraction kit (KeyGen Biotechnology, Nanjing, China). Then, the proteins were quantified, separated, transferred onto PVDF membranes, blocked with 2% bovine serum albumin (BSA, KeyGen Biotechnology) and incubated with the primary antibodies against PRRX2 or GCH1 (Abcam Technology, Cambridge, UK) at 4 °C overnight. After incubation with secondary antibodies (ProteinTech, Chicago, Illinois, USA), the bands were visualized using a chemiluminescence ECL kit (Beyotime Biotechnology, Beijing, China). All results were quantified by ImageJ software (National Institutes of Health, Bethesda, MD, USA).

### Protein stability evaluation

The GSCs were treated with 50 μM proteasome inhibitor MG-132 (Sigma-Aldrich) for 6 h. Then the proteins of GSCs were isolated, and the expression of PRRX2 was detected by western blotting. Furtherly, GSCs were treated with 100 ng/ml cycloheximide (Sigma-Aldrich) for 0, 6, 12, 24, and 36 h, the proteins of GSCs were isolated, and the expression of PRRX2 was also detected by western blotting.

### Ubiquitination assay

First, GSCs were cotransfected with Flag-PRRX2 and HA-UB. Then GSCs were treated with 50 μM MG132 for 6 h and lysed using the total cell protein extraction kit (KeyGen Biotechnology). Ubiquitination of PRRX2 was detected by IP with an antibody against the Flag tag, and then western blotting with an anti-HA antibody (Abcam) was performed.

### MTS assay

MTS assay was performed as previously described [23]. Briefly, GSCs were seeded into 96-well plates at a 1 × 10^3^ cells/ well density for 24, 48, 72, 96, or 120 h. After incubation, 20 μl MTS (3-(4, 5-dimethylthiazol-2-yl)-5-(3-carboxymethoxyphenyl)-2-(4-sulfophenyl)-2H tetrazolium) (Promega, Madison, WI, USA) was added to each well following 3 h incubation at 37 °C. The absorbance at 495 nm was detected using an ultraviolet spectrophotometer (ThermoFisher Scientific, Waltham, MA, USA).

### EdU assay

The 5-ethynyl-20-deoxyuridine (EdU) incorporation assay was performed using an EdU assay kit (Beyotime, Biotechnology) as previously described [23]. Briefly, GSCs were seeded into 24-well plates at 1 × 10^5^ cells/well for 20 h. Then 10 µM EdU reagent was added to each well and continued incubation for 2 h at 37 °C. Finally, the GSCs were photographed using a laser scanning confocal microscope (Olympus), and the percentage of EdU-positive cells was calculated.

### Neurosphere formation assay

The neurosphere formation assay was performed as previously described [23]. Briefly, GSCs were seeded into 24-well plates at 200 cells/well for 7 days. After neurospheres formed, they were photographed under a light microscope (Olympus), and the relative neurosphere sizes were calculated.

### In vitro limiting dilution assay

The in vitro limiting dilution assay was performed as previously described [23]. The GSCs were seeded into 96-well plates at a density of 1, 10, 20, 30, 40, or 50 cells/well, with 10 replicates for each density. Then the neurospheres number was counted after 7 days. The neurosphere synthesis efficiency was calculated via the Extreme Limiting Dilution Analysis (ELDA, http://bioinf.wehi.edu.au/software/elda) [24].

### Lipid ROS detection

The lipid ROS was detected using a BODIPY 581/591 C11 kit (Thermo Fisher Scientific, Waltham, MA, USA). The GSCs were seeded in 6-well plates at 5 × 10^4^ cells/well for 24 h. Then GSCs were stained with 2 μM C11-BODIPY (581/ 591) probe according to the manufacturer’s instructions. GSCs were visualized using a laser scanning confocal microscope (Olympus) and analyzed by Image J software (NIH, Bethesda, MD, USA). The oxidized BODIPY (O-BODIPY) were observed at excitation/emission wavelengths of 488/ 510 (traditional FITC filter set), while the reduced BODIPY (R-BODIPY) were observed at excitation/emission wavelengths of 581/591 nm (Texas Red filter set).

### Glutathione (GSH) assay

GSH was detected using a GSH detection kit (Beyotime Biotechnology). Briefly, GSCs were seeded into 6-well at a 2 × 10^5^ cells/ well density for 24 h. Then GSCs were harvested and resuspended in reagent according to the manufacturer's instructions. The absorbance of products at 412 nm was detected using an ultraviolet spectrophotometer (ThermoFisher Scientific).

### Malondialdehyde (MDA) assay

According to the manufacturer’s instructions, the relative MDA concentration in GSCs was assessed by Lipid Peroxidation Assay Kit (Abcam). Briefly, the GSCs were seeded in 6-well plates at 5 × 10^4^ cells/well for 24 h. Then the GSCs were treated with MDA lysis buffer and reacted with thiobarbituric acid (TBA) to generate an MDA-TBA adduct. The absorbance of the MDA-TBA adduct at 532 nm was detected using an ultraviolet spectrophotometer (ThermoFisher Scientific).

### RNA immunoprecipitation (RIP) assay

RIP assay was performed using an EZ-Magna RIP RNA-binding Protein Immunoprecipitation Kit (Millipore, Darmstadt, Germany) as previously described [23]. Briefly, GSCs were lysed in RIP buffer and incubated with magnetic beads conjugated with anti-PRRX2 antibodies or negative control IgG (Abcam). Then the immunoprecipitated protein-RNAs complex was treated with proteinase K to isolate the RNAs, followed by purification and the circLRFN5 expression in the precipitants was detected by qPCR.

### RNA pull-down assay

The RNA pull-down assay was performed via the Pierce Magnetic RNA Protein pull-down Kit (Thermo Fisher Scientific) as previously described [23]. Briefly, the biotinylated wild or mutant type circLRFN5 probes, the five biotinylated probes containing different fragments of circLRFN5 (△1- 590, △590- 1085, △653- 1085, △1085- 1581 and △1148- 1581) were used to label RNA and pull down the RNA–protein complex. Then the complex was added with magnetic beads and immunoprecipitated. Finally, the proteins were washed, purified and detected by western blotting. β-actin was used as a control.

### Luciferase reporter assay

Luciferase reporter assays were performed as previously described [23]. Briefly, GSCs were seeded into 96-well plates at a density of 5 × 10^3^ cells per well and transfected with the luciferase reporter plasmids of GCH1-wt and GCH1-mt (Gene-Chem) for 48 h. Then the cells were lysed, and luciferase activity was measured using the Dual-Luciferase Reporter Assay System (Promega) according to the manufacturer’s instructions.

### Chromatin immunoprecipitation (ChIP) assays

According to the manufacturer’s instructions, ChIP assays were performed using the ChIP Assay Kit (Beyotime Biotechnology). Briefly, the chromatin complexes were treated with anti-PRRX2 antibody or normal rabbit IgG (Abcam). Then the immunoprecipitated DNA was extracted and purified by qPCR. The primers for ChIP qPCR are listed in Table S3.

### Xenograft experiments

The Xenograft experiments were performed as previously described [10]. Five-week-old female BALB/c nude mice were purchased from Shanghai Jihui Laboratory Animal Care Co., Ltd (Shanghai, China). All mice were bred in the Laboratory Animal Center of Shanghai Tenth People’s Hospital under specific pathogen-free conditions. The animal experiments were performed by the Animal Care Committee of Shanghai Tenth People’s Hospital. Briefly, each group contains five mice, and 5 × 10^4^ GSCs were injected orthotopically into the mouse brain at 2 mm lateral and 2 mm anterior to the bregma with a stereotaxic apparatus. Then the survival time of each mouse was calculated, and the tumor volume was calculated according to the formula: V = (D × d ^2^) / 2, where D represents the longest diameter and d represents the shortest diameter.

### Immunohistochemistry (IHC)

IHC was performed using an immunohistochemical labeling kit (MaxVision Biotechnology, Fuzhou, Fujian, China) as previously described [10]. Briefly, paraffin-embedded sections of tumor specimens were labeled with primary antibodies against PRRX2 (1:100; Abcam), GCH1 (1:100; Abcam), and Ki-67 (1:100; Abcam). The immunohistochemical results were evaluated according to the German immunohistochemical score (GIS) [25].

### Bioinformatics analysis

The basic information of circLRFN5 was obtained from circBase (http://www.circbase.org), Cancer-Specific CircRNA Database (CSCD, http://gb.whu.edu.cn/CSCD/) and circInteractome (https://circinteractome.nia.nih.gov). The binding between circLRFN5 and proteins was predicted via CatRapid (http://service.tartaglialab.com/page/catrapid_group). The ferroptosis-related genes were obtained from Gene set enrichment analysis (GSEA, http:// www.broadinstitute.org/gsea/index.jsp). The expression data of PRRX2 and clinical informations of GBM patients were obtained from the Cancer Genome Atlas (TCGA, http:// cancergenome.nih.gov) in the HG-U133A platform and the Chinese Glioma Genome Atlas (CGGA, http://www.cgga.org.cn) in 325 RNA-seq platform.

### Statistical analysis

The statistical analysis was performed using SPSS 23.0 software (IBM, Armonk, NY, USA) or GraphPad Prism 8.0 (GraphPad Software Inc, San Diego, C.A, USA). All experiments were repeated at least three times, and the results are presented as the mean ± SD. The two-tailed Student’s t-test, chi-square test, or one-way analysis of variance were used to compare the statistical significance among different groups, respectively. The survival curves were plotted by Kaplan–Meier analysis and assessed by log-rank test. The two-tailed *P* < 0.05 was regarded as statistically significant.

## Results

### CircLRFN5 was significantly downregulated in GBM tissues

**Fig. 1:**
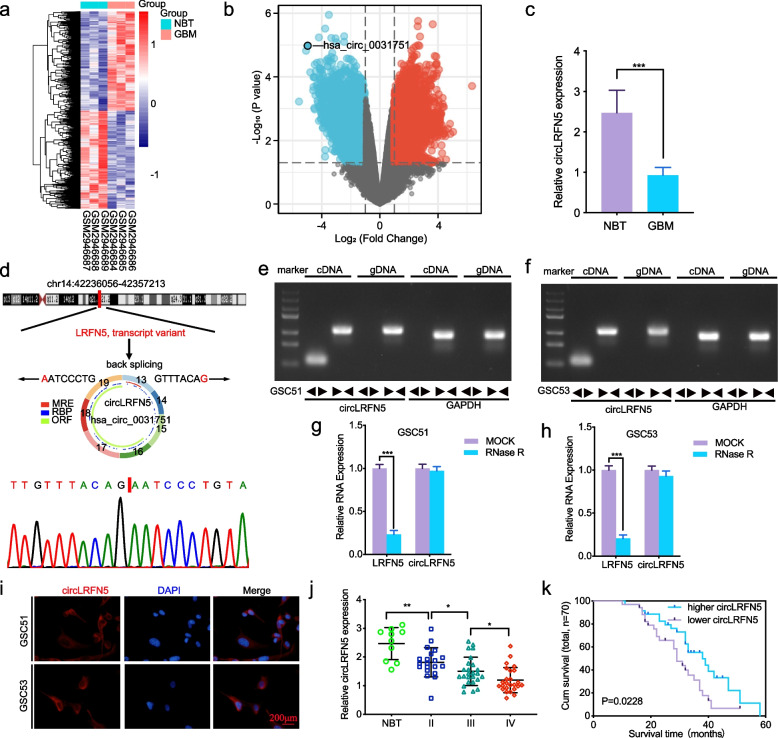
The identification and characteristics of circLRFN5 in GBM. **a**, **b** Heatmaps (**a**) and volcano plots (**b**) of circRNAs that were differentially expressed between GBM and normal brain tissues (NBT) in GSE109569. **c** The expression of circLRFN5 in GBM tissues and NBT as measured by qPCR. **d** Schematic illustration of the genomic location, circular structure exon composition and back splicing site of circLRFN5. The back-splicing site of circLRFN5 was validated by Sanger sequencing. **e**, **f** Agarose gel electrophoresis showed the expression of circLRFN5 in cDNA and gDNA samples from GSC51 (**e**) and GSC53 (**f**) using divergent and convergent primers. β-actin served as a linear RNA control. **g**, **h** RNase treatment was used to evaluate the stability of circLRFN5 and LRFN5 mRNA in GSC51 (**g**) and GSC53 (**h**). **i** FISH assays showed the cellular localization of circLRFN5 in GSC51 and GSC53 cells. Scale bar = 200 μm. **j** The expression of circLRFN5 in 70 different WHO-grade glioma tissues as measured by qPCR. **k** The survival prognosis in different circLRFN5 expression groups was detected in 70 glioma patients. All data are expressed as the mean ± SD (three independent experiments). *p < 0.05; **p < 0.01; ***p < 0.001

At present, most studies on circRNAs focus on their overexpression and roles in GBM. We searched Gene Expression Omnibus (GEO) and found GSE109569 containing circRNA sequencing data between GBM and normal brain tissues [26]. We further analyzed the differentially downregulated circRNAs using the limma R package and found that 1052 circRNAs were downregulated in GBM (Log_2_FC ≤ -2, Padj ≤ 0.05) (Fig. 1a, b). Among the five most downregulated circRNAs, we confirmed their expressions in clinical specimen tissues by qRT-PCR and identified that hsa_circ_0031751 (circLRFN5) was most downregulated in GBM compared with normal brain tissues (Fig. 1c, Fig S1). According to circBase and CSCD, circLRFN5 is an exonic circRNA and back spliced from exon 13 to exon 19 of the LRFN5 transcript with a length of 1581nt (Fig. 1d). Divergent primers were used to amplify the back-splice junction site of circLRFN5, and the result was confirmed by Sanger sequencing (Fig. 1d). Moreover, both divergent and convergent primers were used to amplify circLRFN5, respectively. Agarose gel electrophoresis results showed that circLRFN5 could only be amplified from cDNA by divergent primers (Fig. 1e, f). Besides, RNase R assays showed the circular structure of circLRFN5 was resistant to RNase R treatment without apparent downregulation while its linear RNAs LRFN5 decreased obviously (Fig. 1g, h). FISH assays showed that circLRFN5 was mainly localized to the cytoplasm in GSC51 and GSC53 (Fig. 1i). We furtherly detected its expression in our 70 glioma patients via qRT-PCR. The results showed circLRFN5 expressed lower with increasing WHO grades, and the lowest expression was found in GBM (Fig. 1j). Kaplan–Meier survival analysis showed that the median survival time of patients with lower circLRFN5 expression was shorter than the higher circLRFN5 expression patients (Fig. 1k). In summary, these results indicated that circLRFN5 was significantly downregulated in GBM tissues, and its expression was negatively correlated with GBM patients’ poor prognosis.

### CircLRFN5 inhibits GSCs viability, proliferation, neurospheres formation and stemness

**Fig. 2:**
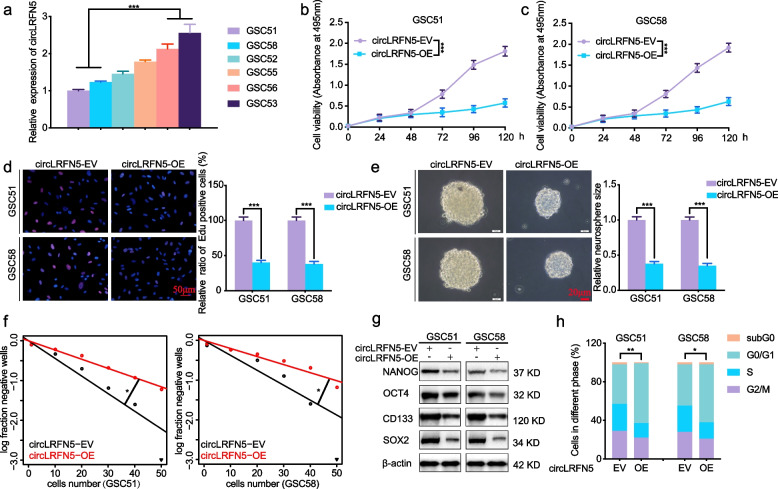
CircLRFN5 inhibits GSCs viability, proliferation, neurospheres formation and stemness. **a** qPCR showed the expression of circLRFN5 in six GBM patients derived GSCs. **b**, **c** MTS assays showed the cell viabilities of GSC51 (**b**) and GSC58 (**c**) after circLRFN5 overexpression. **d** Representive EdU assays showed the proliferation of GSC51 and GSC58 after circLRFN5 overexpression. Scale bar = 50 μm. **e** Representive neurospheres formation assays showed the relative sizes of neurospheres after circLRFN5 overexpression in GSC51 and GSC58. Scale bar = 20 μm. **f** ELDA assays showed the neurospheres formation abilities of GSC51 (left) and GSC58 (right) after circLRFN5 overexpression. **g** Western blotting detects the stemness markers of GSCs (NANOG, OCT4, CD133 and SOX2) after circLRFN5 overexpression in GSC51 and GSC58. **h** Cell cycle assays showed the cell cycle distributions of GSC51 and GSC58 after circLRFN5 overexpression. All data are expressed as the mean ± SD (three independent experiments). *p < 0.05; **p < 0.01; ***p < 0.001

In order to study the possible function of circLRFN5 on GSCs, we cultured six GBM patients derived GSCs, and their circLRFN5 expressions were detected by qPCR. Both GSC51 and GSC58 with lower circLRFN5 expression were chosen for circLRFN5 overexpression, while GSC53 and GSC56 with higher circLRFN5 expression were chosen for circLRFN5 knockdown (Fig. 2a). The efficiencies for circLRFN5 overexpression and knockdown were validated by qPCR (Fig S2a, b). Then MTS assays showed the cell viabilities of GSC51 and GSC58 were obviously decreased after circLRFN5 overexpression (Fig. 2b, c), while the cell viabilities of GSC53 and GSC56 were increased after circLRFN5 knockdown (Fig S3a, b). EdU assays showed the EdU-positive cell rates of GSC51 and GSC58 were also decreased after circLRFN5 overexpression (Fig. 2d) while increased after circLRFN5 knockdown in GSC53 and GSC56 (Fig S3c, d). We also performed neurospheres formation assays and ELDA assays to study the role of circLRFN5 on neurospheres formation abilities. The results showed that the relative neurospheres sizes and neurospheres formation abilities of GSC51 and GSC58 were all decreased after circLRFN5 overexpression (Fig. 2e, f) while increased after circLRFN5 knockdown in GSC53 and GSC56 (Fig S3e, f). We furtherly detected the stemness of GSCs via western blotting. The results showed that the GSCs stemness markers NANOG, OCT4, CD133 and SOX2 decreased after circLRFN5 overexpression in GSC51 and GSC58 (Fig. 2g) while increased after circLRFN5 knockdown in GSC53 and GSC56 (Fig S3g). Moreover, we also performed cell cycle assays and found circLRFN5 overexpression inhibits the G1/S phase transition of GSC51 and GSC58 (Fig. 2h) while promoting the G1/S phase transition of GSC53 and GSC56 after circLRFN5 knockdown (Fig S3h). Taken together, our data suggest that circLRFN5 is functionally important in inhibiting the proliferation and neurospheres formation abilities of GSCs.

### CircLRFN5 overexpression leads to ferroptosis in GSCs

**Fig. 3:**
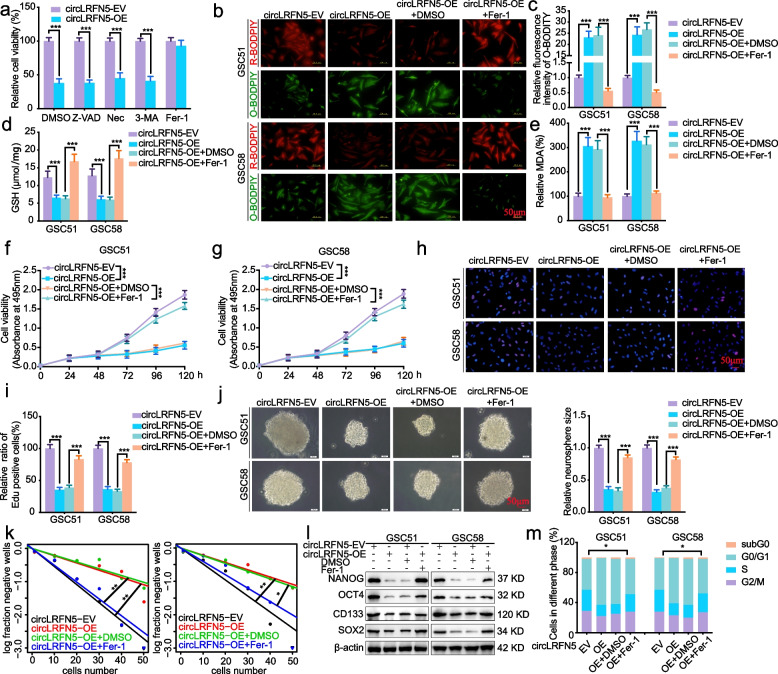
CircLRFN5 inhibits GSCs viability and proliferation in a ferroptosis-dependent manner in vitro. **a** Cell viability assays showed whether VAD (20 μM), Nec (20 μM), 3-MA (60 μM) and Fer-1 (10 μM) could rescue the growth inhibition of GSC51 induced by circLRFN5 overexpression after 48 h. **b**, **c** Representative images of BODIPY (581/591) C11 staining in different groups of GSCs and the relative fluorescence intensity of O-BODIPY was quantified by image J. Scale bar = 50 μm. **d**, **e** GSH (**d**) and MDA (**e**) levels were detected in circLRFN5 overexpressed GSC51 and GSC58, followed by Fer-1 treatment. **f**, **g** MTS assays showed the cell viabilities of circLRFN5 overexpressed GSC51 (**f**) and GSC58 (**g**), followed by Fer-1 treatment. **h**, **i** Representive EdU assays showed the proliferation of circLRFN5 overexpressed GSC51 and GSC58, followed by Fer-1 treatment. Scale bar = 50 μm. **j** Representive neurospheres formation assays showed the relative sizes of neurospheres of circLRFN5 overexpressed GSC51 and GSC58, followed by Fer-1 treatment. Scale bar = 20 μm. **k** ELDA assays showed the neurospheres formation abilities of circLRFN5 overexpressed GSC51 (left) and GSC58 (right), followed by Fer-1 treatment. **l** Western blotting detects the stemness markers of circLRFN5 overexpressed GSC51 and GSC58, followed by Fer-1 treatment. **m** Cell cycle assays showed the cell cycle distributions of circLRFN5 overexpressed GSC51 and GSC58, followed by Fer-1 treatment. All data are expressed as the mean ± SD (three independent experiments). *p < 0.05; **p < 0.01; ***p < 0.001

As the types of cell death are becoming more diversified, the death of tumor cells is not limited to the traditional necrosis and apoptosis, and new types such as ferroptosis and cuproptosis have been discovered one after another. We further discussed the possible mechanism of circLRFN5 on the proliferation and neurospheres formation inhibiting of GSCs. The circLRFN5 overexpressed GSC51 were treated with several cell death inhibitors, including necrostatin-1 (a potent inhibitor of necroptosis), Z-VAD-FMK (a pan-caspase inhibitor), 3-Methyladenine (3-MA, a potent inhibitor of autophagy) and ferrostatin-1 (Fer-1, a potent inhibitor of ferroptosis). The results showed that only Fer-1 treatment could reverse the inhibiting cell viability effect of circLRFN5 (Fig. 3a), indicating that circLRFN5 may inhibit the proliferation and neurospheres formation via promoting ferroptosis.

We furtherly detected whether circLRFN5 overexpression could lead to ferroptosis in GSCs. First, the lipid reactive oxygen species (ROS) accumulation was detected by BODIPY (581/ 591) C11 probe, and the probe bound to peroxidized lipids showed green fluorescence. The results showed that the level of lipid peroxidation was obviously upregulated after circLRFN5 overexpression in GSC51 and GSC58 while downregulated after Fer-1 treatment (Fig. 3b, c). Since glutathione (GSH) depletion is a critical event in ferroptosis, we detected GSH levels and found its level was obviously downregulated after circLRFN5 overexpression while reversed after Fer-1 treatment (Fig. 3d). Moreover, the oxidative stress marker malondialdehyde (MDA) was also detected, and the results showed circLRFN5 overexpression obviously increased the MDA content in GSC51 and GSC58, while reversed after Fer-1 treatment (Fig. 3e). Taken together, these findings strongly suggested that circLRFN5 overexpression can lead to ferroptosis in GSCs.

### CircLRFN5 inhibits GSCs viability and proliferation in a ferroptosis-dependent manner in vitro

We furtherly detected whether circLRFN5 inhibits GSCs viability and proliferation via promoting ferroptosis. All the MTS, EdU, neurospheres formation, ELDA assays and cell cycle assays were performed on the circLRFN5 overexpressed GSC51 and GSC58, followed by Fer-1 treatment. MTS assays (Fig. 3f, g) and EdU assays (Fig. 3h, i) showed the inhibited cell viabilities and proliferation of circLRFN5 overexpressed GSC51 and GSC58 were obviously increased, followed by Fer-1 treatment. The neurospheres formation assays (Fig. 3j) and ELDA assays (Fig. 3k) showed inhibited neurospheres formation abilities of circLRFN5 overexpressed GSC51 and GSC58 recovered after Fer-1 treatment. Western blotting showed the GSCs stemness markers NANOG, OCT4, CD133 and SOX2 decreased after circLRFN5 overexpression in GSC51 and GSC58, while they were all reversed after Fer-1 treatment (Fig. 3l). The cell cycle assays also showed that Fer-1 treatment could promote the G1/S phase transition of circLRFN5 overexpressed GSC51 and GSC58 (Fig. 3m). Therefore, we can conclude that circLRFN5 inhibits GSCs viability and proliferation in a ferroptosis-dependent manner in vitro.

### CircLRFN5 binds to PRRX2 protein in GSCs

**Fig. 4:**
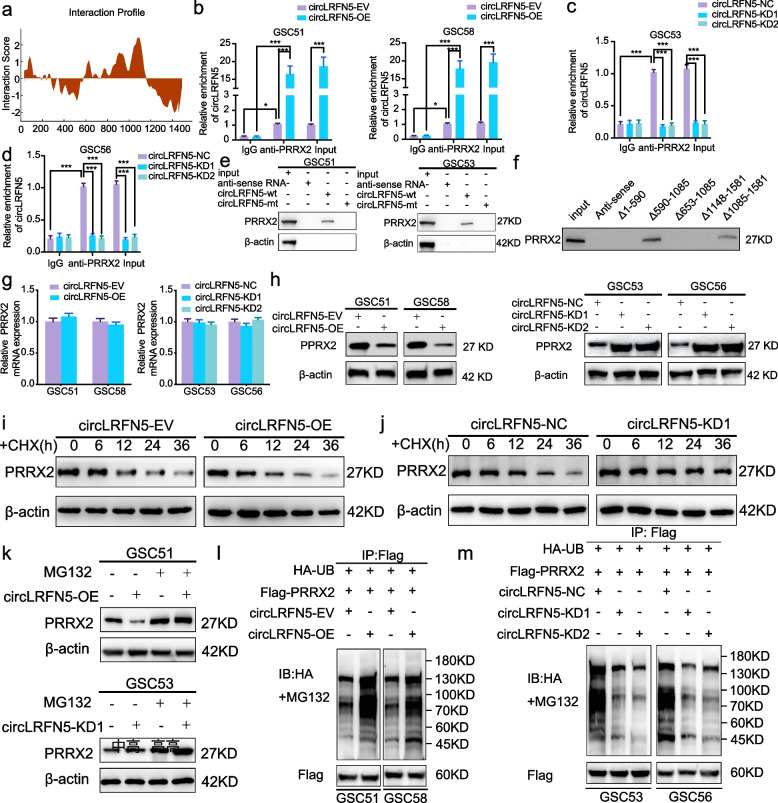
CircLRFN5 binds to PRRX2 protein and promotes its degradation via the ubiquitin-mediated proteasomal pathway. **a** CircLRFN5 binds to PRRX2 proteins via CatRAPID prediction. **b**-**d** RIP assays showed anti-PRRX2 proteins could lead to the enrichment of circLRFN5 in circLRFN5 overexpressed (**b**) or knockdown (**c**,**d**) GSCs. **e** RNA pull-down assays showed the biotinylated circLRFN5 probe pulled down PRRX2 proteins in GSC51 (left) and GSC53 (right). **f** Five biotinylated probes (△1- 590, △590- 1085, △653- 1085, △1085- 1581 and △1148- 1581) containing different fragments of circLRFN5 were designed for RNA pull-down assay. **g** qPCR assays showed the mRNA expression of PRRX2 after circLRFN5 overexpression (left) or knockdown (right) in GSCs. **h** Western blotting showed the protein expression of PRRX2 after circLRFN5 overexpression (left) or knockdown (right) in GSCs. **i**, **j** The circLRFN5 overexpressed GSC51 (**i**) or knockdown GSC53 (**j**) were treated with cycloheximide (CHX, 100 ng/ml) and the half-life of PRRX2 protein was detected by western blotting. **k** The circLRFN5 overexpressed GSC51 (upper) or knockdown GSC53 (lower) was treated with or without MG-132 (50 µM) for 6 h, and PRRX2 expression was detected by western blotting. **l**, **m** In vitro ubiquitination assays showed the level of ubiquitination of PRRX2 protein after circLRFN5 overexpression (**l**) or knockdown (**m**) in GSCs. All data are expressed as the mean ± SD (three independent experiments). *p < 0.05; **p < 0.01; ***p < 0.001

To find the possible downstream mechanism of circLRFN5 induced GSCs ferroptosis and proliferation inhibition, the possible binding proteins of circLRFN5 were predicted by CatRapid. Among the top 120 possible candidate binding proteins, PRRX2 was the only transcription factor (Table S4, Fig. 4a). We performed both RIP and RNA pull-down assays to confirm these binding possibilities. RIP assays found that anti-PRRX2 treatment led to circLRFN5 enrichment compared with IgG treatment. Moreover, higher circLRFN5 enrichment was detected in circLRFN5 overexpressed GSC51 and GSC58 (Fig. 4b), while lower circLRFN5 enrichment was detected in circLRFN5 silenced GSC53 and GSC56 (Fig. 4c, d). The RNA pull-down assays found the wild-type biotinylated circLRFN5 probe pulled down PRRX2 in GSC51 and GSC53, while the mutant type circLRFN5 probe could not (Fig. 4e). In addition, the possible binding region between circLRFN5 and PRRX2 was predicted by CatRapid, and we designed five biotinylated probes containing different fragments of circLRFN5. The following RNA pull-down assays showed that only the probe of △590- 1085 and △1085- 1581 could pull down PRRX2 protein, while the probe of △1- 590, △653- 1085 and △1148- 1581 could not (Fig. 4f). Therefore, we can draw the conclusion that circLRFN5 can bind to PRRX2 at the site of 590–653 nt and 1085 and 1148nt.

### CircLRFN5 promotes PRRX2 degradation via the ubiquitin-mediated proteasomal pathway

We furtherly studied whether circLRFN5 can regulate the expression of PRRX2 in GSCs. First, qPCR assays showed the mRNA level of PRRX2 was almost unchanged after circLRFN5 overexpression or knockdown (Fig. 4g). However, the following western blotting showed the protein levels of circLRFN5 were obviously downregulated after circLRFN5 overexpression while upregulated after circLRFN5 knockdown (Fig. 4h). These results suggested that circLRFN5 may regulate the expression of PRRX2 at the protein level. Then, we performed CHX assays to detect the half-life of PRRX2 protein, and the results showed circLRFN5 overexpression obviously shortened the half-life of PRRX2 in GSC51, while circLRFN5 knockdown increased its half-life in GSC53 (Fig. 4i, j). Besides, the proteasome inhibitor MG-132 was additionally treated with GSCs to inhibit the degradation of PRRX2. The results showed that the reduced protein expression of PRRX2 caused by circLRFN5 overexpression in GSC51 was recovered after MG-132 treatment (Fig. 4k). The protein expression of PRRX2 was even higher in circLRFN5 knockdown GSC53 after MG-132 treatment (Fig. 4k).

Since more than 80% of proteins are degraded via the ubiquitin–proteasome pathway [27], we furtherly studied whether circLRFN5 promotes PRRX2 degradation via a ubiquitin-mediated proteasomal pathway in vitro ubiquitination assay. The results showed circLRFN5 overexpression obviously increased the level of ubiquitination of PRRX2 protein in GSC51 and GSC58, while the ubiquitination level of PRRX2 protein in GSC53 and GSC56 was downregulated after circLRFN5 knockdown (Fig. 4l, m). Taken together, these data suggested that circLRFN5 promotes PRRX2 degradation via a ubiquitin-mediated proteasomal pathway.

### PRRX2 is expressed at higher levels in GBM and is correlated with poor patient survival

**Fig. 5:**
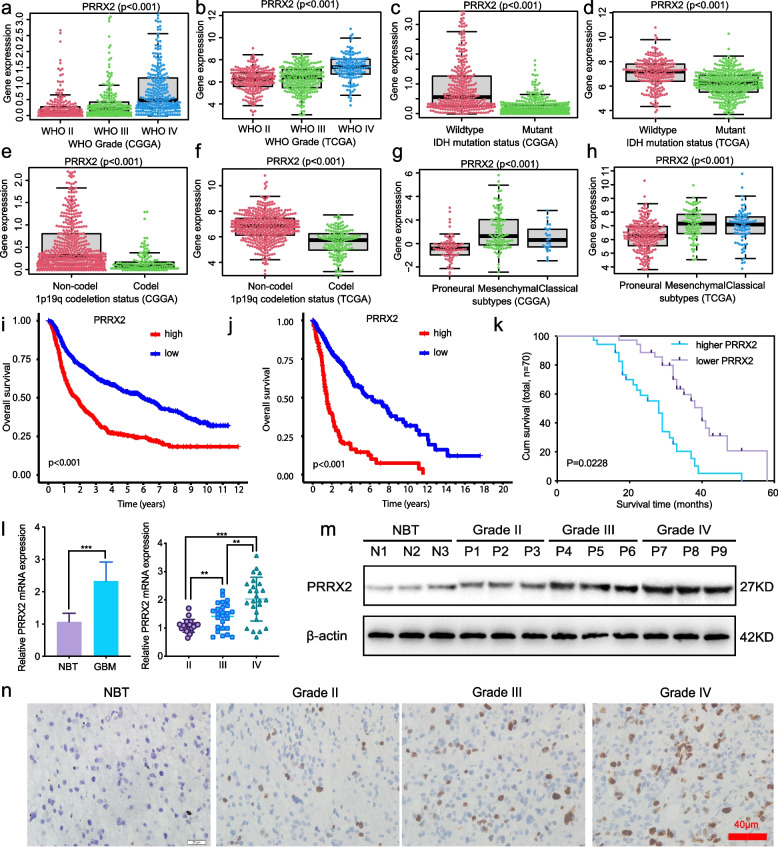
PRRX2 is expressed at higher levels in GBM and is correlated with poor patient survival. **a**, **c**, **e**, **g** The expression of PRRX2 in different WHO grades (**a**), IDH mutant status (**c**), 1p19q codeletion status (**e**) and three molecular subtypes (**g**) in CGGA GBM datasets. **b**, **d**, **f**, **h** The expression of PRRX2 in different WHO grades (**b**), IDH mutant status (**d**), 1p19q codeletion status (**f**) and three molecular subtypes (**g**) in TCGA GBM datasets. **i**-**k** The prognostic significance of PRRX2 was confirmed in the CGGA (**i**), TGGA **(j**) databases and our 70 glioma patients (**k**) according to Kaplan–Meier survival analysis. **l**: qPCR assays showed the mRNA expression of PRRX2 in GBM and NBT tissues (left) and different WHO-grade glioma tissues (right). **m**, **n** Western blotting (**m**) and immunohistochemistry assays (**n**) showed PRRX2 expression in different WHO-grade glioma tissues. Scale bar = 40 μm. All data are expressed as the mean ± SD (three independent experiments). *p < 0.05; **p < 0.01; ***p < 0.001

Although we confirmed that circLRFN5 binds to and promotes PRRX2 degradation via the ubiquitin-mediated proteasomal pathway, there is no study on the expression and function of PRRX2 in GBM. We first analyzed its expression in CGGA and TCGA GBM datasets. The results showed PPRX2 expressed at a higher level in higher WHO grade (Fig. 5a, b), wild-type IDH status (Fig. 5c, d), 1p19q non-codeletion status (Fig. 5e, f) and mesenchymal subtypes (Fig. 5g, h) than the lower WHO grade, mutant type IDH status, 1p19q codeletion status and proneural or classical subtypes, respectively. Besides, Kaplan–Meier survival analysis showed that higher PRRX2 expression patients' median survival time was shorter than patients with lower PRRX2 expression in CGGA and TCGA GBM datasets (Fig. 5i, j). We furtherly validated its expression in our 70 glioma patients. All the qPCR (Fig. 5l), western blotting (Fig. 5m) and immunohistochemistry assays (Fig. 5n) confirmed PRRX2 expressed higher in glioma and GBM tissues than the NBT and even higher with increasing WHO grades, and the highest expression was found in GBM. The Kaplan–Meier survival analysis showed that the median survival time of patients with higher PRRX2 expression was shorter than the lower PRRX2 expression patients (Fig. 5k). In summary, these results indicated that PRRX2 was significantly upregulated in GBM tissues, and its expression was positively correlated with GBM patients’ poor prognosis.

### PRRX2 promotes GSCs viability and proliferation

Since PRRX2 is overexpressed in GBM and correlates with poor prognosis, we furtherly studied whether PRRX2 promotes GSCs viability and proliferation. Both the MTS assays (Fig. S4a, b) and EdU assays (Fig. S4c, d) showed that PRRX2 overexpression obviously promoted the viability and proliferation of GSC51 and GSC58. Then neurospheres formation assays (Fig. S4e) and ELDA assays (Fig. S4f) showed that PRRX2 overexpression promoted the neurospheres formation abilities of GSC51 and GSC58. The cell cycle assays also showed that PRRX2 overexpression promoted the G1/S phase transition of GSC51 and GSC58 (Fig. S4g). These results confirmed the promoting cell viability, proliferation and neurospheres formation effect of PRRX2 overexpression on GSCs.

### PRRX2 promotes GSCs viability and proliferation via inhibiting ferroptosis

Furtherly, we also detected the level of ferroptosis in PRRX2 overexpressed GSCs. The BODIPY (581/ 591) C11 assays showed that the level of lipid peroxidation was obviously downregulated after PRRX2 overexpression in GSC51 and GSC58, while upregulated after the ferroptosis activator RSL3 treatment (Fig. S4h, i). The GSH levels were obviously upregulated after PRRX2 overexpression while downregulated after RSL3 treatment (Fig. S4j). Moreover, the MDA level in PRRX2 overexpressed GSC51 and GSC58 was obviously decreased while increased after RSL3 treatment (Fig. S4k). Taken together, these findings strongly suggested that PRRX2 overexpression can inhibit ferroptosis in GSCs. Then, all the MTS MTS, EdU, neurospheres formation, ELDA assays and cell cycle assays were performed on RSL3 treated PRRX2 overexpressed GSC51 and GSC58 again. The results showed that RSL3 treatment could obviously reverse the promoting cell viability, proliferation, neurospheres formation, and the G1/S phase transition of PRRX2 overexpressed GSC51 and GSC58. Therefore, we can conclude that PRRX2 promotes GSCs viability and proliferation via inhibiting ferroptosis.

### CircLRFN5 inhibits GSCs viability and proliferation and promotes ferroptosis via PRRX2 degradation

Since circLRFN5 can lead to ubiquitin-mediated PRRX2 degradation and PRRX2 promotes GSCs viability and proliferation via inhibiting ferroptosis, we furtherly studied whether PRRX2 was the essential downstream gene of circLRFN5 mediated GSCs inhibition. MTS assays (Fig. S5a, b) and EdU assays (Fig. S5c, d) showed the inhibited cell viabilities and proliferation of circLRFN5 overexpressed GSC51 and GSC58 were obviously increased combined with PRRX2 overexpression. The neurospheres formation assays (Fig. S5e) and ELDA assays (Fig. S5f) showed inhibited neurospheres formation abilities of circLRFN5 overexpressed GSC51 and GSC58 were also recovered after PRRX2 overexpression. The cell cycle assays also showed that PRRX2 overexpression could promote the G1/S phase transition of circLRFN5 overexpressed GSC51 and GSC58 (Fig. S5g).

Moreover, we also detected PRRX2 overexpression on the level of ferroptosis of circLRFN5 overexpressed GSCs. The BODIPY (581/ 591) C11 assays showed the level of lipid peroxidation was obviously upregulated after circLRFN5 overexpression in GSC51 and GSC58, while downregulated after PRRX2 overexpression (Fig. S5h, i). The GSH levels were downregulated after circLRFN5 overexpression while reversed after PRRX2 overexpression (Fig. S5j). Besides, the MDA content in circLRFN5 overexpressed GSC51 and GSC58 was decreased while reversed after PRRX2 overexpression (Fig. S5k). Taken together, these findings strongly suggested that circLRFN5 overexpression can inhibit GSCs viability and proliferation and lead to ferroptosis in GSCs via PRRX2 degradation.

### PRRX2 transcriptionally upregulates GCH1 expression in GSCs

**Fig. 6:**
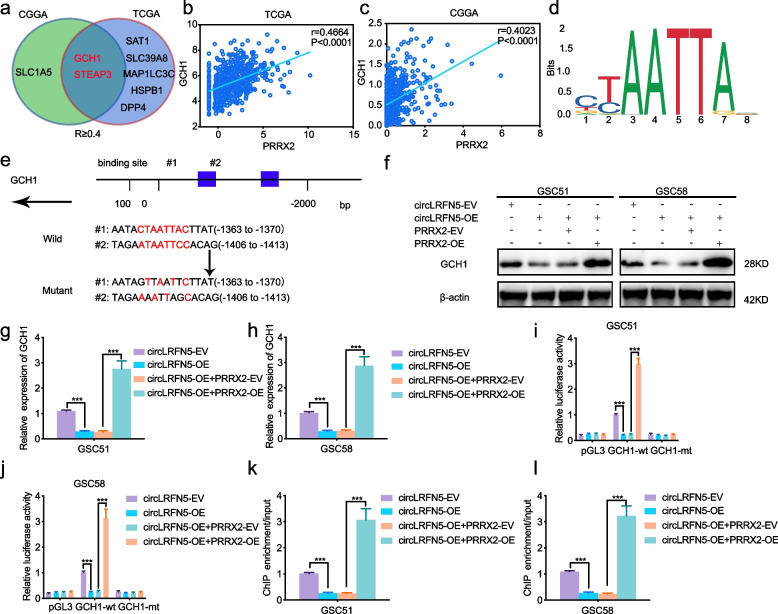
CircLRFN5 downregulates PRRX2 mediated transcription of GCH1 in GSCs. **a** A Venn Diagram showed the correlation between PRRX2 and ferroptosis-related genes in TCGA and CGGA datasets (R more than 0.4). **b**, **c** The correlation between PRRX2 and GCH1 in TCGA (**b**) and CGGA (**c**) datasets. **d** Sequence motif representing the consensus PRRX2 binding motif according to the JASPAR database. **e** Schematic diagram of the putative PRRX2 binding site in the promoter of GCH1. **f**–**h** Western blotting (**f**) and qPCR (**g**,** h**) showed the expression of GCH1 in circLRFN5 overexpressed GSC51 and GSC58, followed by PRRX2 overexpression. **i**, **j** The luciferase reporter assays showed the luciferase promoter activities of GCH1 after circLRFN5 overexpression in GSC51 (**i**) and GSC58 (**j**), followed by PRRX2 overexpression. **k**, **l** The ChIP qPCR showed that anti-PRRX2 treatment could lead to the enrichment of GCH1 in circLRFN5 overexpressed GSC51 (**k**) and GSC58 (**l**), followed by PRRX2 overexpression. All data are expressed as the mean ± SD (three independent experiments). *p < 0.05; **p < 0.01; ***p < 0.001

Considering there is no study about the function of PRRX2 on ferroptosis, we first analyzed the correlation between PRRX2 and ferroptosis-related genes in TCGA and CGGA datasets. The detailed results are shown in Table S5. Only two candidate genes (GCH1 and STEAP3) were found to be negatively correlated with PRRX2, with a correlation coefficient of more than 0.4 (Fig. 6a-c). Then, we detected the expression of GCH1 and STEAP3 after PRRX2 overexpression or knockdown via qPCR. The results showed that the expression of STEAP3 was almost unchanged after PRRX2 regulation (Fig. S6a, b), while the expression of GCH1 was obviously upregulated after PRRX2 overexpression, while downregulated after PRRX2 knockdown (Fig. S6c, d). The following western blotting also confirmed the regulation of PRRX2 on GCH1 (Fig. S6e, f).

Since PRRX2 was a transcription factor (Fig. 6d), we furtherly analyzed the possible transcription and binding sites of PRRX2 on the promoter of GCH1 via the JASPAR database (Fig. 6e). Then, we designed the luciferase reporter assays and the results showed that the relative luciferase activity of pGL3-GCH1-wt was obviously upregulated in PRRX2 overexpressed GSC51 and GSC58 (Fig. S6g, h), while downregulated after PRRX2 knockdown in GSC53 and GSC56 (Fig. S6i, j). Finally, ChIP assays also showed that anti-PRRX2 treatment could increase enrichment of GCH1 after PRRX2 overexpression in GSC51 and GSC58, while decreased enrichment of GCH1 after enrichment PRRX2 knockdown in GSC53 and GSC56 (Fig. S6k, l). In summary, these results demonstrated that PRRX2 transcriptionally upregulates GCH1 expression in GSCs.

### CircLRFN5 downregulates PRRX2 mediated transcription of GCH1 in GSCs

We furtherly studied whether circLRFN5 can regulate the expression of GCH1 via PRRX2. Both western blotting (Fig. 6f) and qPCR (Fig. 6g, h) showed circLRFN5 overexpression could obviously downregulate GCH1 expression in GSC51 and GSC58, while was reversed and even upregulated obviously combined with PRRX1 overexpression. Then the relative luciferase activity of pGL3-GCH1-wt was obviously downregulated in circLRFN5 overexpressed GSC51 and GSC58, while upregulated following PRRX2 overexpression (Fig. 6i, j). Besides, the ChIP assays also showed that anti-PRRX2 treatment could lead to decreasing enrichment of GCH1 after circLRFN5 overexpression in GSC51 and GSC58, while it was reversed following PRRX2 overexpression (Fig. 6k, l). Therefore, these results demonstrate that circLRFN5 can downregulate PRRX2-mediated transcription of GCH1 in GSCs.

### CircLRFN5 promotes ferroptosis via down-regulating GCH1 in GSCs

**Fig. 7:**
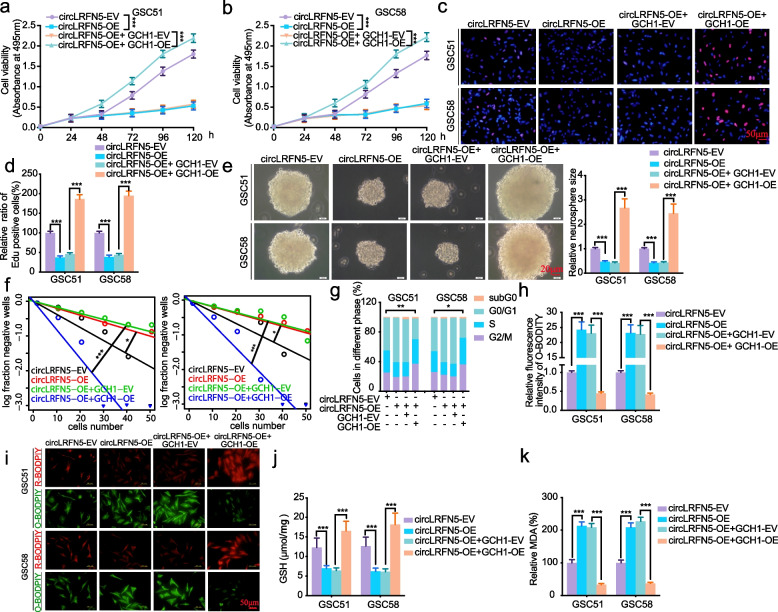
CircLRFN5 promotes ferroptosis via down-regulating GCH1 in GSCs. **a**, **b** MTS assays showed the cell viabilities of circLRFN5 overexpressed GSC51 (**a**) and GSC58 (**b**) after GCH1 overexpression. **c**, **d** Representative images of EdU assays showed the proliferation of circLRFN5 overexpressed GSC51 and GSC58, followed by GCH1 overexpression. Scale bar = 50 μm. **e** Representative images of neurospheres formation assays showed the relative sizes of neurospheres of circLRFN5 overexpressed GSC51 and GSC58, followed by GCH1 overexpression. Scale bar = 20 μm. **f** ELDA assays showed the neurospheres formation abilities of circLRFN5 overexpressed GSC51 (left) and GSC58 (right), followed by GCH1 overexpression. **g** Cell cycle assays showed the cell cycle distributions of circLRFN5 overexpressed GSC51 and GSC58, followed by GCH1 overexpression. **h**, **i** Representative images of BODIPY (581/591) C11 staining in circLRFN5 overexpressed GSC51 and GSC58, followed by GCH1 overexpression. The relative fluorescence intensity of O-BODIPY was quantified by image J. Scale bar = 50 μm. **j**, **k** GSH (**j**) and MDA (**k**) levels were detected in circLRFN5 overexpressed GSC51 and GSC58, followed by GCH1 overexpression. All data are expressed as the mean ± SD (three independent experiments). *p < 0.05; **p < 0.01; ***p < 0.001

GCH1 was reported as a ferroptosis suppressor via generating antioxidant tetrahydrobiopterin (BH4) [28] and regulated GBM growth and tumor-initiating cell maintenance [29]. It is worth studying whether circLRFN5 regulates the proliferation, neurospheres formation and ferroptosis via GCH1 in GSCs. First, MTS assays (Fig. 7a, b) and EdU assays (Fig. 7c, d) showed the inhibited cell viabilities and proliferation of circLRFN5 overexpressed GSC51 and GSC58 were also increased combined with GCH1 overexpression. The neurospheres formation assays (Fig. 7e) and ELDA assays (Fig. 7f) showed circLRFN5 overexpression inhibited neurospheres formation abilities of GSC51 and GSC58 were also recovered after GCH1 overexpression. The cell cycle assays also showed that circLRFN5 overexpression inhibited G1/S phase transition was reversed after GCH1 overexpression in GSC51 and GSC58 (Fig. 7g).

We furtherly detected the level of ferroptosis of circLRFN5 overexpressed GSCs after GCH1 overexpression. The BODIPY (581/ 591) C11 assays showed the level of lipid peroxidation was obviously upregulated after circLRFN5 overexpression in GSC51 and GSC58, while downregulated after GCH1 overexpression (Fig. 7h, i). The GSH levels in circLRFN5 overexpressed GSC51 and GSC58 were obviously downregulated after circLRFN5 overexpression, while reversed after GCH1 overexpression (Fig. 7j). Finally, the MDA content was obviously increased in circLRFN5 overexpressed GSC51 and GSC58, while reversed after GCH1 overexpression (Fig. 7k). These rescue experiments indicated that the inhibiting GSCs proliferation, neurospheres formation and inducing ferroptosis effects of circLRFN5 was depended on down-regulating GCH1.

### CircLRFN5 regulates tumorigenesis of GSCs in vivo

**Fig. 8:**
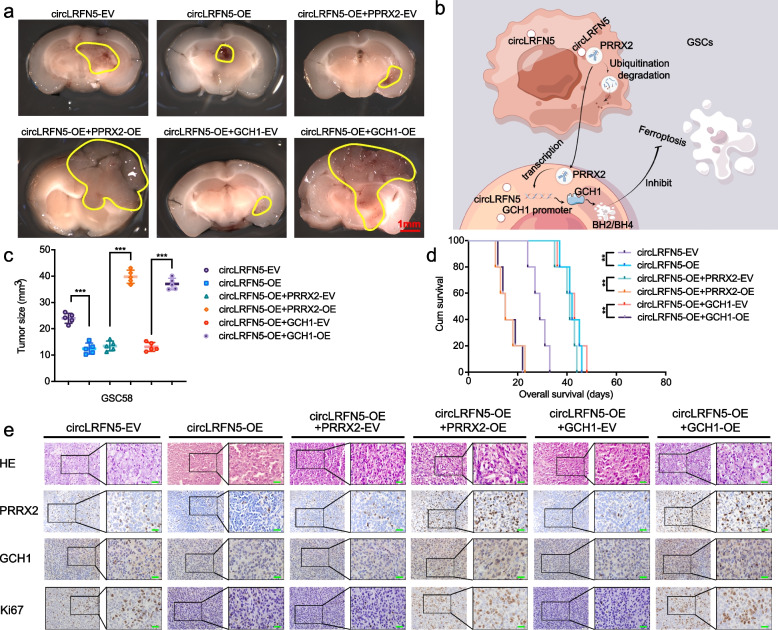
CircLRFN5 regulates tumorigenesis of GSCs in vivo. **a**, **c** Representative photographs showed the sizes of intracranial tumors in the coronal position of the vector control group, circLRFN5 overexpressed group and combined with the PRRX2 or GCH1 overexpression group. Scale bar = 10 mm. **b** Schematic diagram showed circLRFN5 inhibits GSCs viability, proliferation and tumorigenesis and promotes ferroptosis via PRRX2 degradation and GCH1 downregulation (http://www.figdraw.com). **d** Kaplan–Meier survival curves showed the survival times of vector control group, circLRFN5 overexpressed group and combined with PRRX2 or GCH1 overexpression group. **e** Representative immunohistochemical images showed the staining and expression of PRRX2, GCH1 and Ki-67 of the vector control group, circLRFN5 overexpressed group and combined with PRRX2 or GCH1 overexpression group. Scale bar = 50 μm. All data are expressed as the mean ± SD (three independent experiments). *p < 0.05; **p < 0.01; ***p < 0.001

In order to demonstrate the GBM suppressive effects of circLRFN5 in vivo, we performed the orthotopic xenograft experiments using GSC51 to evaluate the effects of circLRFN5 on GBM tumorigenesis. The tumor volumes and sizes were obviously decreased after circLRFN5 overexpression, while they increased after PRRX2 overexpression or GCH1 overexpression, respectively (Fig. 8a, c). Kaplan–Meier survival analysis showed the median survival time of the circLRFN5 overexpressed group was longer than the empty vector group, while accompanied by shorter survival times in circLRFN5 overexpression combined with PRRX2 overexpression or GCH1 overexpression groups (Fig. 8d). Moreover, IHC was performed to detect the effects of circLRFN5 on the staining intensity and expression levels of Ki-67, PRRX2 and GCH1. The results showed circLRFN5 overexpression obviously decreased their expression, while the staining intensity and expression levels of Ki-67, PRRX2 and GCH1 were reversed and even upregulated combined with PRRX2 overexpression or GCH1 overexpression (Fig. 8e). Taken together, the schematic diagram showed that circLRFN5 inhibits GSCs viability, proliferation and tumorigenesis and promotes ferroptosis via PRRX2 degradation and GCH11 downregulation (Fig. 8b).

## Discussion

In recent years, increasing studies have found that various circRNAs are abnormally expressed in GBM, which are involved in the occurrence, development and malignant progression of GBM [30]. CircASAP1 expression was significantly upregulated in GBM and promotes tumorigenesis and temozolomide resistance of GBM via NRAS/MEK1/ERK1-2 signaling [31]. CircHEATR5B encoded a novel protein and suppressed aerobic glycolysis of GBM through phosphorylation of JMJD5 [32]. The circular RNA-encoded oncogenic e-cadherin variant promotes GBM tumorigenicity by activating the EGFR-STAT3 signal [33]. With the development of circRNA sequencing and molecular biology technologies, an increasing number of novel circRNAs involved in the occurrence and progression of GBM have been found. Therefore, searching for GBM-related circRNAs helps understand the molecular mechanism of GBM occurrence and progression and provides molecular targeted therapeutic targets for GBM.

Our study found that a novel circRNA, circLRFN5, was downregulated in GBM compared with normal brain tissues, and its expression was negatively correlated with GBM patients' poor prognosis. Then we detected its biological functions in GSCs and orthotopic xenograft models. We found that circLRFN5 could inhibit GSCs viability, proliferation, neurospheres formation, stemness, and tumorigenesis in vivo. Therefore, circLRFN5 was considered to play a suppressive role in GBM.

Ferroptosis is a new type of programmed cell death (PCD) found in recent years, which is iron-dependent [34]. Ferroptosis is different from other cell death such as necrosis, apoptosis and autophagy in cell morphology, mechanism and biochemistry [35]. The biochemical characteristics of ferroptosis mainly include the accumulation of iron and ROS in cells, the activation of MAPKs signal transduction system, the inhibition of the cystine/ glutamate transporter system and the increase of NADPH oxidation [36]. Ferroptosis was reported to play an anti-cancer effect in many kinds of malignant tumors and acted as a critical regulator of tumor growth [12]. In GBM, RNA binding protein NKAP protects tumor cells from ferroptosis by promoting SLC7A11 mRNA splicing in an m6A-dependent manner [37]. Inhibition of autophagy increases the susceptibility of GBM stem cells to temozolomide by igniting ferroptosis [38].

Our study found that circLRFN5 overexpression can lead to the accumulation of lipid ROS and MDA and glutathione depletion. Then, the ferroptosis inhibitor treatment (Fer-1) could reverse the inhibiting GSCs effect of circLRFN5. Especially, Fer-1 treatment can even recover the expression of stemness markers in circLRFN5 overexpressed GSCs. Several studies have reported the possible relationships between ferroptosis and GSCs. For example, RSL3 induced GSCs differentiation into astrocytes, which was accompanied by the downregulation of NESTIN and SOX2 via the TGM2/AKT/ID1 signaling axis [39]. The stem cell factor SOX2 can inhibit ferroptosis in lung cancer stem-like cells via upregulating SLC7A11 expression [40]. Therefore, our study indicated that circLRFN5 inhibits GSCs viability, proliferation and stemness in ferroptosis-dependent.

Although many studies have discovered the detailed mechanism and critical signaling pathways of ferroptosis, the mechanism of ncRNAs, especially circRNAs, in the occurrence and progression of ferroptosis is still unclear [19]. Only a few studies have revealed the role of circRNAs in ferroptosis and GBM. It was reported that circCDK14 promotes tumor progression and resists ferroptosis in glioma by regulating PDGFRA [16]. CircTTBK2 regulates cell proliferation, invasion, and ferroptosis via glioma's miR-761/ITGB8 axis [41]. In our study, to find the possible downstream of circLRFN5 mediated ferroptosis in GSCs, we first found a transcription factor, PRRX2, that circLRFN5 can directly bind. Moreover, we found circLRFN5 could promote degradation of PRRX2 via ubiquitin-mediated proteasomal pathway.

PRRX2 was reported as an oncogene in several cancers, including hepatocellular carcinoma, prostate cancer, and colon cancer [21, 22, 42]. PRRX2 enhances invasion and migration in mammary epithelial cells and correlates with poor prognosis in breast cancer via activating TGF-β [43]. However, there was no study on its expression and biological functions in GBM and GSCs. Our study demonstrated its overexpression in GBM tissues according to bioinformatics analysis on TCGA and CGGA datasets and validation of clinical GBM specimens. Moreover, we found its expression is positively correlated with GBM patients’ poor prognosis, and PRRX2 overexpression could promote GSCs viability, proliferation, and neurospheres formation via inhibiting ferroptosis. Therefore, we discovered that PRRX2 is a novel oncogene in GBM and promoted malignant phenotypes of GSCs via inhibiting ferroptosis. Moreover, it is worth exploring whether circLRFN5 induces ferroptosis by promoting ubiquitin-mediated degradation of PRRX2. The following rescue experiments confirmed this conclusion. Our experiment results showed that PRRX2 overexpression can obviously inhibit the ferroptosis caused by circLRFN5 overexpression and even promotes the proliferation and neurospheres formation of GSCs.

Although the above studies demonstrate that circLRFN5 mediated PRRX2 degradation participates in the ferroptosis of GSCs, the directly targeted ferroptosis-related genes are still unknown. We first got the ferroptosis-related genes via GSEA. Then the correlation between PRRX2 and ferroptosis-related genes in TCGA and CGGA datasets was analyzed. Therefore, we found two candidate ferroptosis-related genes (STEAP3 and GCH1) with a correlation coefficient of more than 0.4. Moreover, the following qPCR and western blotting confirmed that PRRX2 could regulate only GCH1. Besides, since PRRX2 was a transcription factor, we furtherly studied whether PRRX2 can transcriptionally unregulate GCH1. Both the luciferase reporter assays and ChIP assays confirmed this conclusion. Furtherly, the rescue experiments demonstrated that circLRFN5 downregulates GCH1 expression in GSCs via PRRX2 degradation.

GCH1 is also called GTP cyclohydrolase-1 and is a novel oncogene participating in suppressive ferroptosis [44]. Tetrahydrobiopterin/ dihydrobiopterin (BH4/BH2) is the metabolic derivative of GCH1 [28]. BH4/BH2 can further lead to lipid remodeling and suppress ferroptosis by selectively preventing the depletion of phospholipids with two polyunsaturated fatty acyl tails [45]. Moreover, GCH1 was found to be overexpressed in GBM, promoted growth, maintained the brain tumor-initiating cells and suppressed the reactive oxygen species in GBM [29]. Therefore, GCH1 is a candidate downstream gene of circLRFN5 since it plays a crucial role in the pro-tumorigenic of GBM via suppressing ferroptosis. Our experiments furtherly demonstrated that GCH1 overexpression could reverse the inhibiting effects of circLRFN5 overexpression on GSCs via inhibiting ferroptosis.

## Conclusion

In summary, our study found that a novel circRNA circLRFN5 was down-expressed in GBM, and its low expression was correlated with patients’ poor prognosis. CircLRFN5 played a suppressive role in GSCs, inhibiting GSCs viability, proliferation, neurospheres formation, stemness and tumorigenesis and promoting ferroptosis. Mechanistically, circLRFN5 binds to PRRX2 protein and promotes its degradation via the ubiquitin-mediated proteasomal pathway, while PRRX2 transcriptionally upregulates GCH1 expression in GSCs. Therefore, circLRFN5 downregulates PRRX2 mediated transcription of GCH1 in GSCs. GCH1 is responsible for suppressing ferroptosis and promoting the malignant phenotypes of GSCs. Our study identified the role of circLRFN5 in the progression of ferroptosis and GBM. CircLRFN5 can be used as a potential GBM biomarker and become a target for molecular therapies or ferroptosis-dependent therapy in GBM.

## Supplementary Information

**Additional file 1:** **Supplementary Figure 1. **Validation of the expression of the top five downregulated circRNAs in GBM and NBT tissues. **a** The detailed Log_2_FC and Padj value of the top five downregulated circRNAs in GSE109569 using limma R package. **b** The expression of these top five downregulated circRNAs in GBM tissues and NBT as measured by qPCR. All data are expressed as the mean ± SD (three independent experiments). *p < 0.05; **p < 0.01; ***p < 0.001.

**Additional file 2:** **Supplementary Figure 2. **The expression of circLRFN5, PRRX2, and GCH1 in GSCs after lentiviral-based transfection. **a, b** qPCR showed circLRFN5 expression after circLRFN5 overexpression in GSC51 and GSC58 (**a**) or knockdown in GSC53 and GSC56 (**b**). **c, d** qPCR (**c**) and western blotting (**d**) showed PRRX2 expression after PRRX2 overexpression in GSC51 and GSC58. **e, f** qPCR (**e**) and western blotting (**f**) showed GCH1 expression after GCH1 overexpression in GSC51 and GSC58. All data are expressed as the mean ± SD (three independent experiments). *p < 0.05; **p < 0.01; ***p < 0.001.

**Additional file 3:** **Supplementary Figure 3. **CircLRFN5 silencing promotes GSCs viability, proliferation, neurospheres formation and stemness. **a, b** MTS assays showed the cell viabilities of GSC56 (**a**) and GSC53 (**b**) after circLRFN5 knockdown. **c, d** Representive EdU assays showed the proliferation of GSC53 and GSC56 after circLRFN5 knockdown. Scale bar = 50 μm. e Representive neurospheres formation assays showed the relative sizes of neurospheres after circLRFN5 knockdown in GSC53 and GSC56. Scale bar = 20 μm. **f** ELDA assays showed the neurospheres formation abilities of GSC53 (left) and GSC56 (right) after circLRFN5 knockdown. g Western blotting detects the stemness markers of GSCs after circLRFN5 knockdown in GSC53 and GSC56. **h** Cell cycle assays showed the cell cycle distributions of GSC53 and GSC56 after circLRFN5 knockdown. All data are expressed as the mean ± SD (three independent experiments). *p < 0.05; **p < 0.01; ***p < 0.001.

**Additional file 4:** **Supplementary Figure 4. **PRRX2 promotes GSCs viability and proliferation via inhibiting ferroptosis. **a, b** Cell viability assays showed the cell viabilities of GSC51 (**a**) and GSC58 (**b**) after PRRX2 overexpression, followed by RSL3 treatment. **c, d** Representative images of EdU assays showed the proliferation of PRRX2 overexpressed GSC51 and GSC58, followed by RSL3 treatment. Scale bar = 50 μm. **e** Representative images of neurospheres formation assays showed the relative sizes of neurospheres of PRRX2 overexpressed GSC51 and GSC58, followed by RLS3 treatment. Scale bar = 20 μm. **f** ELDA assays showed the neurospheres formation abilities of PRRX2 overexpressed GSC51 (left) and GSC58 (right), followed by RSL3 treatment. **g** Cell cycle assays showed the cell cycle distributions of PRRX2 overexpressed GSC51 and GSC58, followed by RSL3 treatment. **h, i** Representative images of BODIPY (581/591) C11 staining in PRRX2 overexpressed GSC51 and GSC58, followed with RSL3 treatment. The relative fluorescence intensity of O-BODIPY was quantified by image J. Scale bar = 50 μm. **j, k** GSH (j) and MDH (k) levels were detected in PRRX2 overexpressed GSC51 and GSC58, followed by RSL3 treatment. All data are expressed as the mean ± SD (three independent experiments). *p < 0.05; **p < 0.01; ***p < 0.001

**Additional file 5:** **Supplementary Figure 5. **CircLRFN5 inhibits GSCs viability and proliferation and promotes ferroptosis via PRRX2 degradation. **a, b** Cell viability assays showed the cell viabilities of circLRFN5 overexpressed GSC51 (a) and GSC58 (b) after PRRX2 overexpression. **c, d** Representative images of EdU assays showed the proliferation of circLRFN5 overexpressed GSC51 and GSC58, followed by PRRX2 overexpression. Scale bar = 50 μm. **e** Representative images of neurospheres formation assays showed the relative sizes of neurospheres of circLRFN5 overexpressed GSC51 and GSC58, followed by PRRX2 overexpression. Scale bar = 20 μm. **f** ELDA assays showed the neurospheres formation abilities of circLRFN5 overexpressed GSC51 (left) and GSC58 (right), followed by PRRX2 overexpression. **g** Cell cycle assays showed the cell cycle distributions of circLRFN5 overexpressed GSC51 and GSC58, followed by PRRX2 overexpression. **h, i** Representative images of BODIPY (581/591) C11 staining in circLRFN5 overexpressed GSC51 and GSC58, followed by PRRX2 overexpression. The relative fluorescence intensity of O-BODIPY was quantified by image J. Scale bar = 50 μm. **j, k** GSH (j) and MDH (k) levels were detected in circLRFN5 overexpressed GSC51 and GSC58, followed by PRRX2 overexpression. All data are expressed as the mean ± SD (three independent experiments). *p < 0.05; **p < 0.01; ***p < 0.001.

**Additional file 6:** **Supplementary Figure 6. **PRRX2 transcriptionally upregulates GCH1 expression in GSCs. **a-d** qPCR showed the mRNA expression of STEAP3 (**a, b**) and GCH1 (**c, d**) in PRRX2 overexpressed GSC51 and GSC58 or PRRX2 knockdown GSC53 and GSC56. **e, f** Western blotting showed the expression of GCH1 in PRRX2 overexpressed (**e**) or knockdown (**f**) GSCs. **g-j** The luciferase reporter assays showed the luciferase promoter activities of GCH1 after PRRX2 overexpression in GSC51 (**g**) and GSC58 (**h**) or PRRX2 knockdown in GSC53 (**i**) and GSC56 (**j**). **k, l** The ChIP qPCR showed that anti-PRRX2 treatment could enrich GCH1 in PRRX2 overexpressed (**k**) or knockdown (**l**) GSCs. All data are expressed as the mean ± SD (three independent experiments). *p < 0.05; **p < 0.01; ***p < 0.001.

**Additional file 7:** **Supplementary Table 1.** Clinical information of the primary glioma stem cells.

**Additional file 8:** **Supplementary Table 2. **siRNA sequences.

**Additional file 9:** **Supplementary Table 3. **Primers for qRT-PCR and ChIP.

**Additional file 10:** **Supplementary Table 4.** The interaction between circLRFN5 and top 120 proteins.

**Additional file 11:** **Supplementary Table 5**. Correlation between PPRX2 and ferropotsis related genes in TCGA and CGGA datasets.

## Data Availability

The analyzed data sets generated during the present study are available from the corresponding author on reasonable request.

## Abbreviations

NBT: Normal brain tissues
GSCs: Glioma stem cells
GBM: Glioblastoma
circRNAs: Circular RNAs
GSH: Glutathione
MDA: Malondialdehyde
PUFAs: Polyunsaturated fatty acids
L-OOH: Lipid hydroperoxide
PRRX2: Paired related homeobox 2
TF: Transcription factor
RBPs: RNA binding proteins
qRT-PCR: Real-time quantitative reverse transcription PCR
RIP: RNA immunoprecipitation
ChIP: Chromatin immunoprecipitation
ELISA: Enzyme-linked immunosorbent assay
IHC: Immunohistochemistry
MTS: 3-(4, 5-Dimethylthiazol-2-yl)-5-(3-carboxymethoxyphenyl)-2-(4-sulfophenyl)-2H tetrazolium
EdU: 5-Ethynyl-20-deoxyuridine
ELDA: Extreme Limiting Dilution Analysis
BH4: Tetrahydrobiopterin
ncRNAs: Non-coding RNAs
RCD: Regulated cell death
ROS: Reactive oxygen species
WHO: World Health Organization
STR: Short tandem repeat
O-BODIPY: Oxidized BODIPY
R-BODIPY: Reduced BODIPY
TBA: Thiobarbituric acid
GIS: German immunohistochemical score
CSCD: Cancer-Specific CircRNA Database
GSEA: Gene set enrichment analysis
TCGA: The Cancer Genome Atlas
CGGA: Chinese Glioma Genome Atlas
GEO: Gene Expression Omnibus
PCD: Programmed cell death
