# Correction: CircLRFN5 inhibits the progression of glioblastoma via PRRX2/GCH1 mediated ferroptosis

**DOI:** 10.1186/s13046-022-02527-7

**Published:** 2022-11-10

**Authors:** Yang Jiang, Junshuang Zhao, Rongqing Li, Yingliang Liu, Lin Zhou, Chengbin Wang, Caihong Lv, Liang Gao, Daming Cui

**Affiliations:** 1 Department of Neurosurgery, Shanghai Tenth People’s Hospital, TongjiUniversity School of Medicine, Shanghai, 200072 China; 2 Department of Neurosurgery, Taihe Affiliated Hospital of Hubei University of Medicine, Shiyan, 442000 China

**Correction: *J Exp Clin Cancer Res* 41, 307 (2022)**

**https://doi.org/10.1186/s13046-022-02518-8**

Following publication of the original article [1], author identified an error in Fig. 4k. It was caused by errors in the publishing process. The correct figure is presented below:

**Fig. 4:**
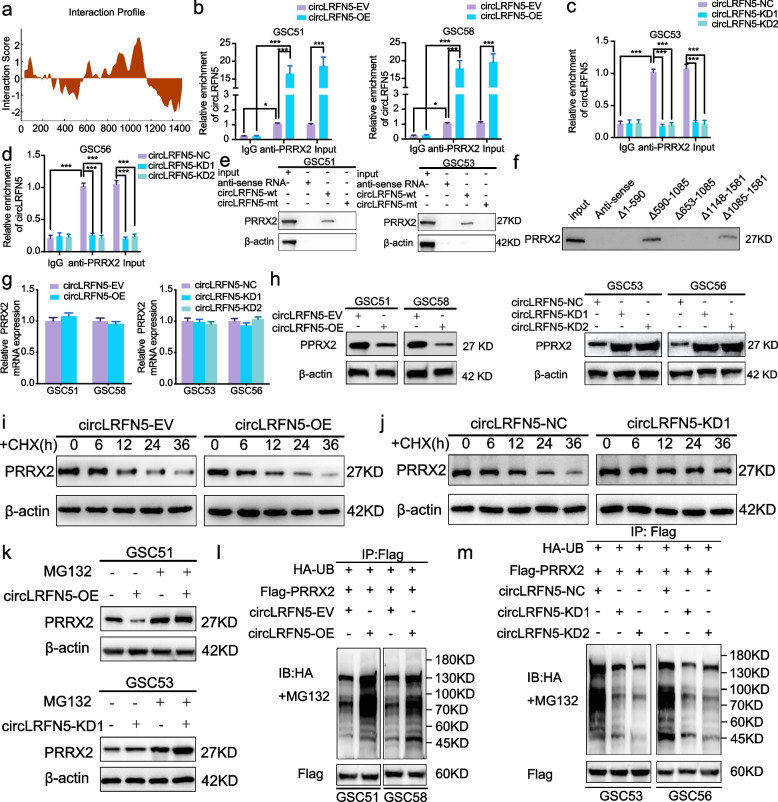
CircLRFN5 binds to PRRX2 protein and promotes its degradation via the ubiquitin-mediated proteasomal pathway. **a** CircLRFN5 binds to PRRX2 proteins via CatRAPID prediction. **b**-**d** RIP assays showed anti-PRRX2 proteins could lead to the enrichment of circLRFN5 in circLRFN5 overexpressed (**b**) or knockdown (**c**,**d**) GSCs. **e** RNA pull-down assays showed the biotinylated circLRFN5 probe pulled down PRRX2 proteins in GSC51 (left) and GSC53 (right). **f** Five biotinylated probes (△1- 590, △590- 1085, △653- 1085, △1085- 1581 and △1148- 1581) containing different fragments of circLRFN5 were designed for RNA pull-down assay. **g** qPCR assays showed the mRNA expression of PRRX2 after circLRFN5 overexpression (left) or knockdown (right) in GSCs. **h** Western blotting showed the protein expression of PRRX2 after circLRFN5 overexpression (left) or knockdown (right) in GSCs. **i**, **j** The circLRFN5 overexpressed GSC51 (**i**) or knockdown GSC53 (**j**) were treated with cycloheximide (CHX, 100 ng/ml) and the half-life of PRRX2 protein was detected by western blotting. **k** The circLRFN5 overexpressed GSC51 (upper) or knockdown GSC53 (lower) was treated with or without MG-132 (50 μM) for 6 h, and PRRX2 expression was detected by western blotting. **l**, **m** In vitro ubiquitination assays showed the level of ubiquitination of PRRX2 protein after circLRFN5 overexpression (**l**) or knockdown (**m**) in GSCs. All data are expressed as the mean ±SD (three independent experiments). *p < 0.05; **p < 0.01; ***p < 0.001

The correction does not have any effect on the results or conclusions of the paper. The original article has been corrected.
