# Supplementary figures and images for: CircLRFN5 inhibits the progression of glioblastoma via PRRX2/GCH1 mediated ferroptosis
Source: J Exp Clin Cancer Res. 2022 Oct 20;41:307. doi: 10.1186/s13046-022-02518-8 (PMC9583503; doi:10.1186/s13046-022-02518-8)

a

| ID               | logFC      | AveExpr    | t          | P.adj      |
|------------------|------------|------------|------------|------------|
| hsa_circ_0083682 | -5.5709726 | 10.2718034 | -6.1861616 | 0.00060315 |
| hsa_circ_0017169 | -5.0891032 | 3.77274303 | -11.441168 | 1.51E-05   |
| hsa_circ_0031751 | -4.9750868 | 4.25672825 | -12.123892 | 1.05E-05   |
| hsa_circ_0027581 | -4.7975834 | 3.98269564 | -8.8774874 | 7.20E-05   |
| hsa_circ_0035024 | -4.5998243 | 10.4360579 | -7.1131316 | 0.00026989 |

b

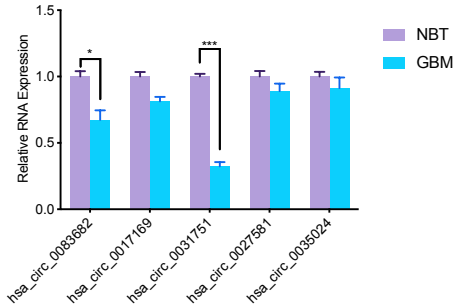

Supplement: Supplementary file 1 — Additional file 1: Supplementary Figure 1. Validation of the expression of the top five downregulated circRNAs in GBM and NBT tissues. a The detailed Log2FC and Padj value of the top five downregulated circRNAs in GSE109569 using limma R package. b The expression of these top five downregulated circRNAs in GBM tissues and NBT as measured by qPCR. All data are expressed as the mean ± SD (three independent experiments). *p < 0.05; **p < 0.01; ***p < 0.001. [file 13046_2022_2518_MOESM1_ESM.pdf]

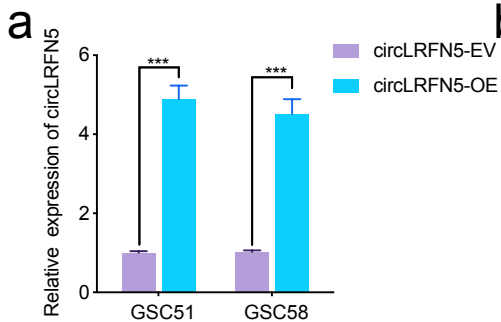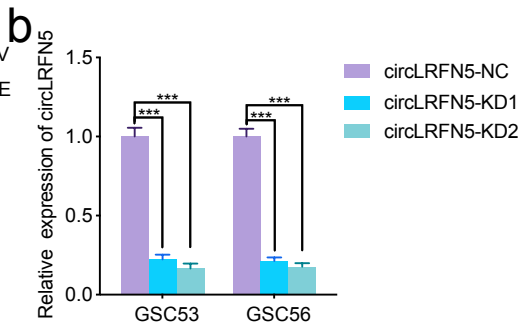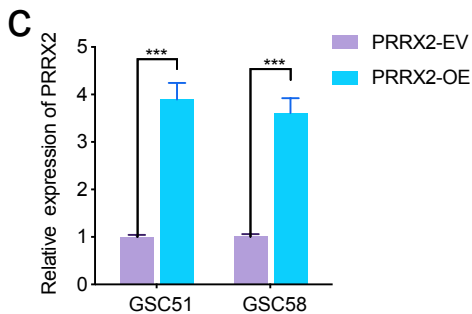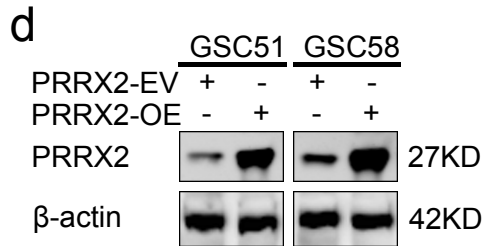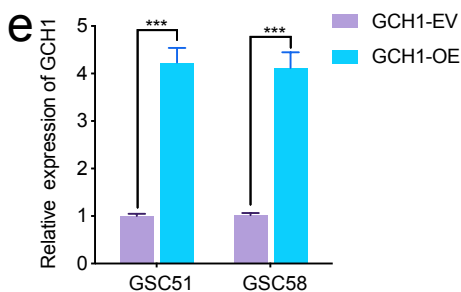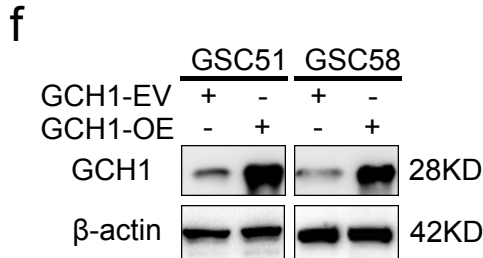

Supplement: Supplementary file 2 — Additional file 2: Supplementary Figure 2. The expression of circLRFN5, PRRX2, and GCH1 in GSCs after lentiviral-based transfection. a, b qPCR showed circLRFN5 expression after circLRFN5 overexpression in GSC51 and GSC58 (a) or knockdown in GSC53 and GSC56 (b). c, d qPCR (c) and western blotting (d) showed PRRX2 expression after PRRX2 overexpression in GSC51 and GSC58. e, f qPCR (e) and western blotting (f) showed GCH1 expression after GCH1 overexpression in GSC51 and GSC58. All data are expressed as the mean ± SD (three independent experiments). *p < 0.05; **p < 0.01; ***p < 0.001. [file 13046_2022_2518_MOESM2_ESM.pdf]

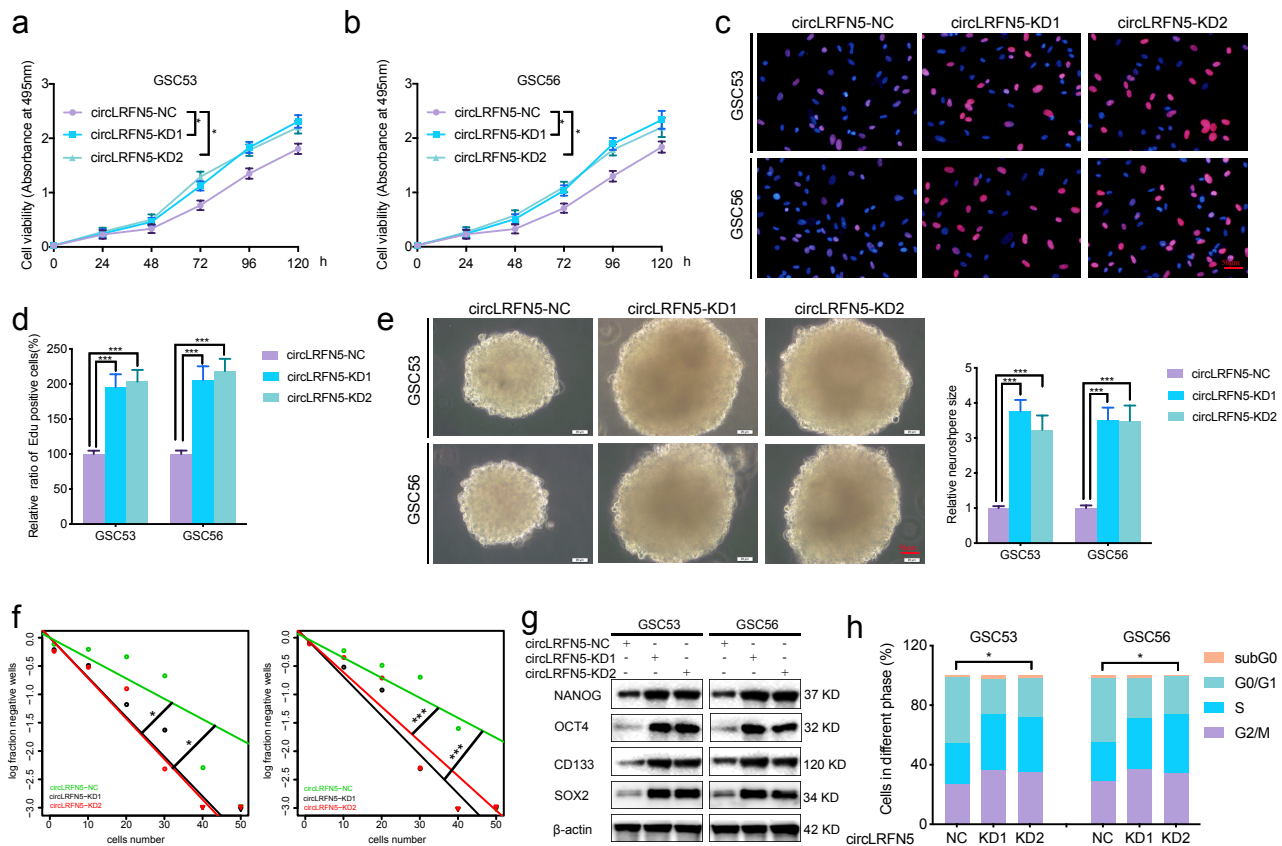

Supplement: Supplementary file 3 — Additional file 3: Supplementary Figure 3. CircLRFN5 silencing promotes GSCs viability, proliferation, neurospheres formation and stemness. a, b MTS assays showed the cell viabilities of GSC56 (a) and GSC53 (b) after circLRFN5 knockdown. c, d Representive EdU assays showed the proliferation of GSC53 and GSC56 after circLRFN5 knockdown. Scale bar = 50 μm. e Representive neurospheres formation assays showed the relative sizes of neurospheres after circLRFN5 knockdown in GSC53 and GSC56. Scale bar = 20 μm. f ELDA assays showed the neurospheres formation abilities of GSC53 (left) and GSC56 (right) after circLRFN5 knockdown. g Western blotting detects the stemness markers of GSCs after circLRFN5 knockdown in GSC53 and GSC56. h Cell cycle assays showed the cell cycle distributions of GSC53 and GSC56 after circLRFN5 knockdown. All data are expressed as the mean ± SD (three independent experiments). *p < 0.05; **p < 0.01; ***p < 0.001. [file 13046_2022_2518_MOESM3_ESM.pdf]

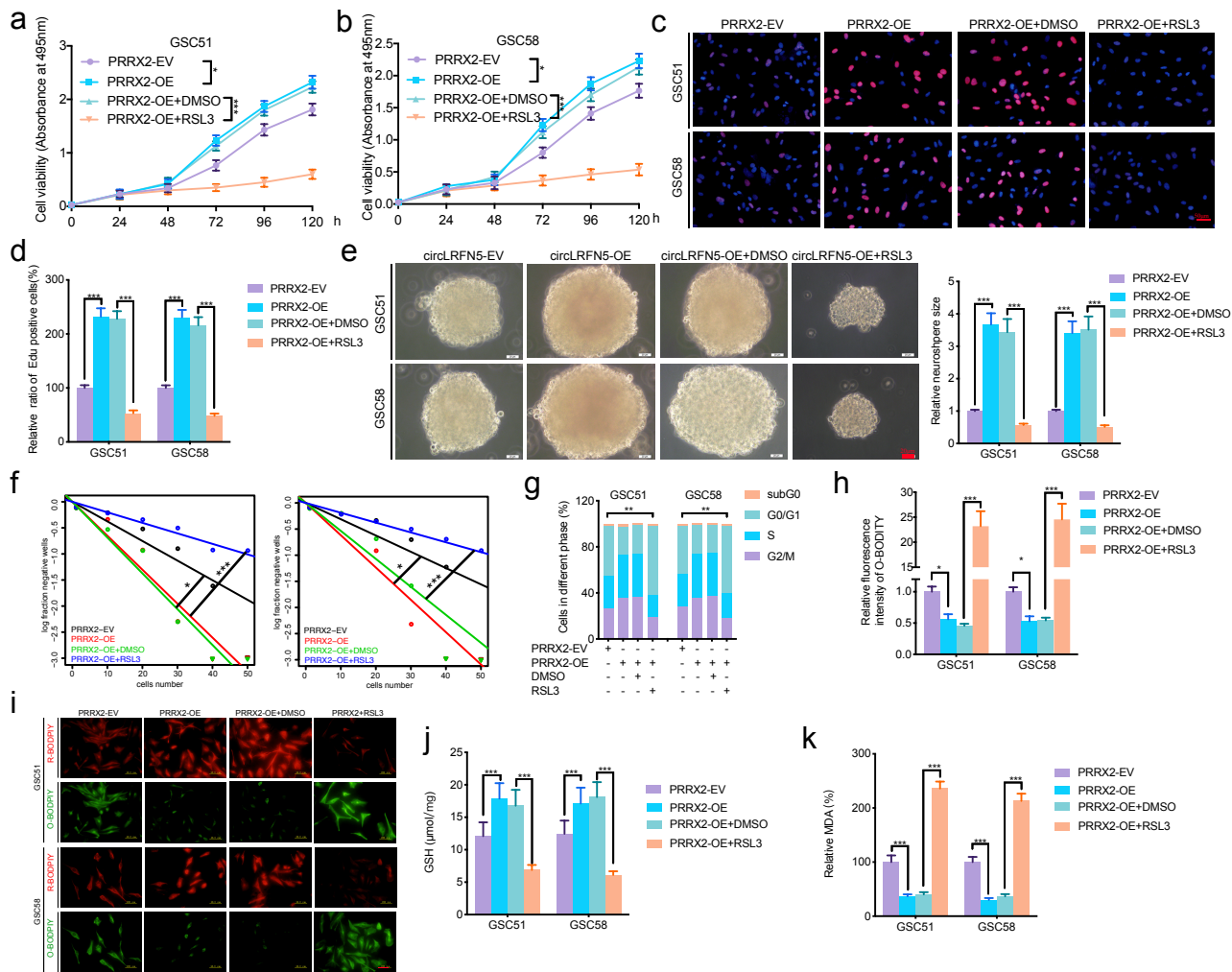

Supplement: Supplementary file 4 — Additional file 4: Supplementary Figure 4. PRRX2 promotes GSCs viability and proliferation via inhibiting ferroptosis. a, b Cell viability assays showed the cell viabilities of GSC51 (a) and GSC58 (b) after PRRX2 overexpression, followed by RSL3 treatment. c, d Representative images of EdU assays showed the proliferation of PRRX2 overexpressed GSC51 and GSC58, followed by RSL3 treatment. Scale bar = 50 μm. e Representative images of neurospheres formation assays showed the relative sizes of neurospheres of PRRX2 overexpressed GSC51 and GSC58, followed by RLS3 treatment. Scale bar = 20 μm. f ELDA assays showed the neurospheres formation abilities of PRRX2 overexpressed GSC51 (left) and GSC58 (right), followed by RSL3 treatment. g Cell cycle assays showed the cell cycle distributions of PRRX2 overexpressed GSC51 and GSC58, followed by RSL3 treatment. h, i Representative images of BODIPY (581/591) C11 staining in PRRX2 overexpressed GSC51 and GSC58, followed with RSL3 treatment. The relative fluorescence intensity of O-BODIPY was quantified by image J. Scale bar = 50 μm. j, k GSH (j) and MDH (k) levels were detected in PRRX2 overexpressed GSC51 and GSC58, followed by RSL3 treatment. All data are expressed as the mean ± SD (three independent experiments). *p < 0.05; **p < 0.01; ***p < 0.001 [file 13046_2022_2518_MOESM4_ESM.pdf]

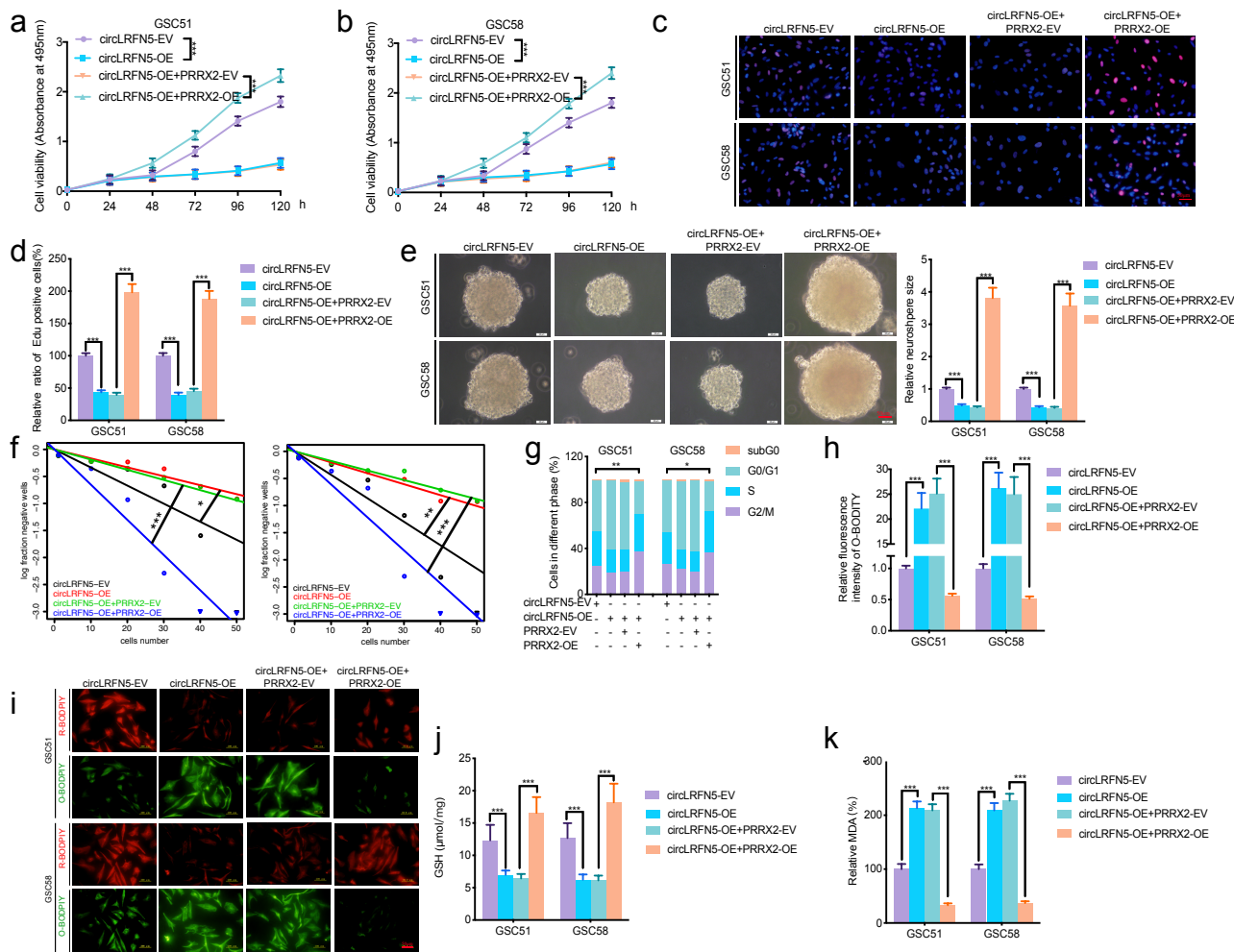

Supplement: Supplementary file 5 — Additional file 5: Supplementary Figure 5. CircLRFN5 inhibits GSCs viability and proliferation and promotes ferroptosis via PRRX2 degradation. a, b Cell viability assays showed the cell viabilities of circLRFN5 overexpressed GSC51 (a) and GSC58 (b) after PRRX2 overexpression. c, d Representative images of EdU assays showed the proliferation of circLRFN5 overexpressed GSC51 and GSC58, followed by PRRX2 overexpression. Scale bar = 50 μm. e Representative images of neurospheres formation assays showed the relative sizes of neurospheres of circLRFN5 overexpressed GSC51 and GSC58, followed by PRRX2 overexpression. Scale bar = 20 μm. f ELDA assays showed the neurospheres formation abilities of circLRFN5 overexpressed GSC51 (left) and GSC58 (right), followed by PRRX2 overexpression. g Cell cycle assays showed the cell cycle distributions of circLRFN5 overexpressed GSC51 and GSC58, followed by PRRX2 overexpression. h, i Representative images of BODIPY (581/591) C11 staining in circLRFN5 overexpressed GSC51 and GSC58, followed by PRRX2 overexpression. The relative fluorescence intensity of O-BODIPY was quantified by image J. Scale bar = 50 μm. j, k GSH (j) and MDH (k) levels were detected in circLRFN5 overexpressed GSC51 and GSC58, followed by PRRX2 overexpression. All data are expressed as the mean ± SD (three independent experiments). *p < 0.05; **p < 0.01; ***p < 0.001. [file 13046_2022_2518_MOESM5_ESM.pdf]

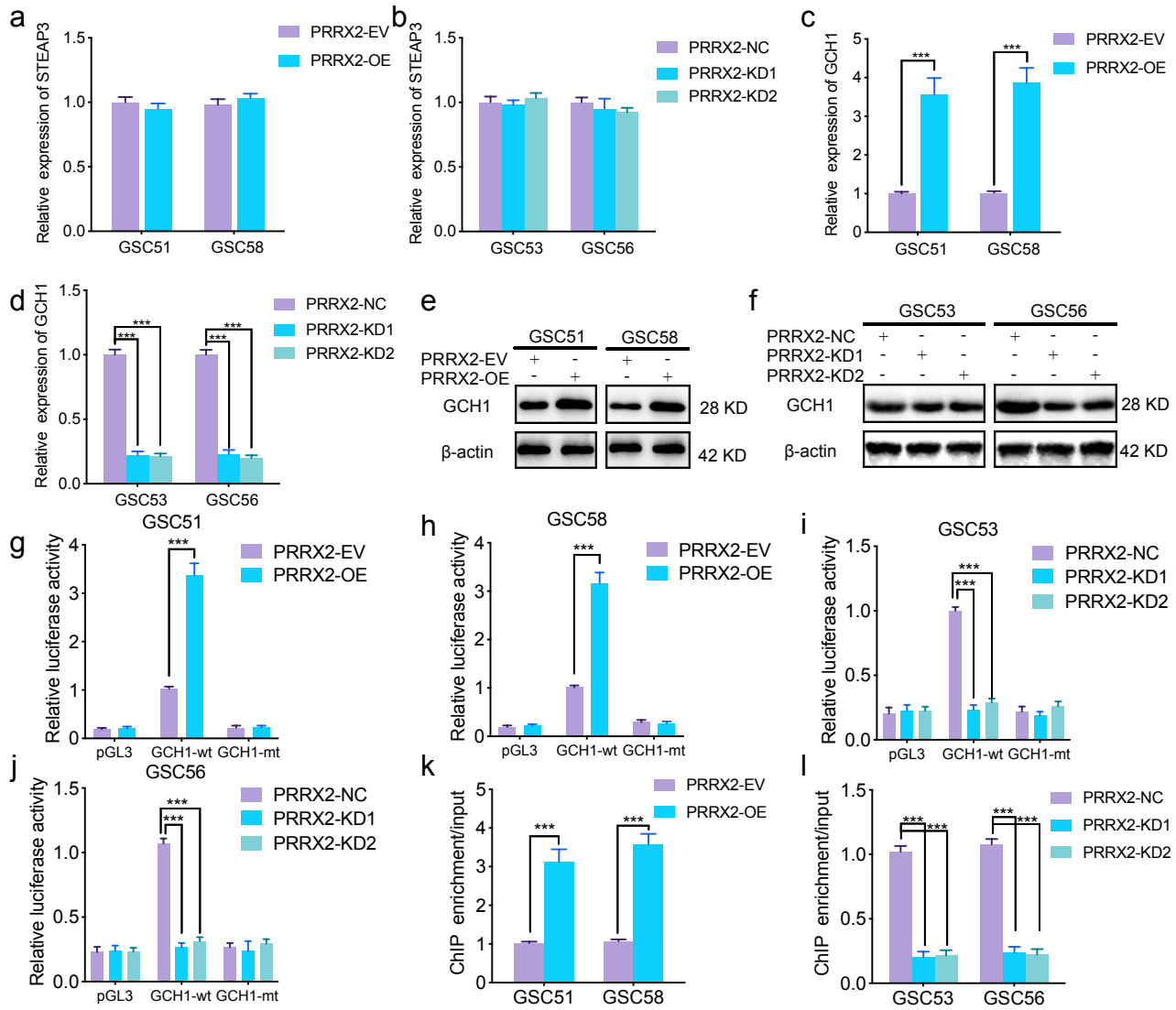

Supplement: Supplementary file 6 — Additional file 6: Supplementary Figure 6. PRRX2 transcriptionally upregulates GCH1 expression in GSCs. a-d qPCR showed the mRNA expression of STEAP3 (a, b) and GCH1 (c, d) in PRRX2 overexpressed GSC51 and GSC58 or PRRX2 knockdown GSC53 and GSC56. e, f Western blotting showed the expression of GCH1 in PRRX2 overexpressed (e) or knockdown (f) GSCs. g-j The luciferase reporter assays showed the luciferase promoter activities of GCH1 after PRRX2 overexpression in GSC51 (g) and GSC58 (h) or PRRX2 knockdown in GSC53 (i) and GSC56 (j). k, l The ChIP qPCR showed that anti-PRRX2 treatment could enrich GCH1 in PRRX2 overexpressed (k) or knockdown (l) GSCs. All data are expressed as the mean ± SD (three independent experiments). *p < 0.05; **p < 0.01; ***p < 0.001. [file 13046_2022_2518_MOESM6_ESM.pdf]
