# Supplementary material for: CircLRFN5 inhibits the progression of glioblastoma via PRRX2/GCH1 mediated ferroptosis
Source: J Exp Clin Cancer Res. 2022 Oct 20;41:307. doi: 10.1186/s13046-022-02518-8 (PMC9583503; doi:10.1186/s13046-022-02518-8)
Supplement: Supplementary file 7 — Additional file 7: Supplementary Table 1. Clinical information of the primary glioma stem cells. [file 13046_2022_2518_MOESM7_ESM.docx]

Supplementary Table 1. Clinical information of the primary glioma stem cells.

|  | GSC51 | GSC52 | GSC53 | GSC55 | GSC56 | GSC58 |
| --- | --- | --- | --- | --- | --- | --- |
| Gender | Male | Male | Female | Female | Female | Male |
| Age | 51 years old | 63 years old | 48 years old | 41 years old | 53 years old | 46 years old |
| Location | Left insula | Left frontal lobe | Right occipital lobe | Right frontal lobe | Right occipital lobe | Left insula |
| Pathological diagnosis | Glioblastoma | Glioblastoma | Glioblastoma | Glioblastoma | Glioblastoma | Glioblastoma |
| WHO grade | IV | IV | IV | IV | IV | IV |
| Ki-67 | 20% (+) | 20% (+) | 55% (+) | 35% (+) | 30% (+) | 20% (+) |
| IDH status | Wild | Wild | Wild | Wild | Wild | Wild |
| 1p/19q status | Non-codeletion | Non-codeletion | Non-codeletion | Non-codeletion | Non-codeletion | Non-codeletion |
| H3F3A status | Mutant | Mutant | Mutant | Mutant | Mutant | Mutant |
| MGMT status | Unmethylation | Unmethylation | Unmethylation | Unmethylation | Unmethylation | Unmethylation |
