# Supplementary material for: CircLRFN5 inhibits the progression of glioblastoma via PRRX2/GCH1 mediated ferroptosis
Source: J Exp Clin Cancer Res. 2022 Oct 20;41:307. doi: 10.1186/s13046-022-02518-8 (PMC9583503; doi:10.1186/s13046-022-02518-8)
Supplement: Supplementary file 8 — Additional file 8: Supplementary Table 2. siRNA sequences. [file 13046_2022_2518_MOESM8_ESM.docx]

| **Primer** | **Forward (5’-3’)** | **Reverse (5’-3’)** |
| --- | --- | --- |
| circLRFN5-KD1 | AUCAUAAGUACCAUUGUACUG | GUACAAUGGUACUUAUGAUGA |
| circLRFN5-KD2 | AUUGUAGAUCAGGUUUAAGAG | CUUAAACCUGAUCUACAAUGG |
| PRRX2-KD1 | AUACACAAACUAAAUGAGGUC | CCUCAUUUAGUUUGUGUAUUA |
| PRRX2-KD2 | AAUACACAAACUAAAUGAGGU | CUCAUUUAGUUUGUGUAUUAA |
| siRNA-NC | UUCUUCGAAGGUGUCACGUTT | ACGUGACACCUUCGAAGAATT |

**Supplementary Table 2. siRNA sequences**
