# Supplementary material for: CircLRFN5 inhibits the progression of glioblastoma via PRRX2/GCH1 mediated ferroptosis
Source: J Exp Clin Cancer Res. 2022 Oct 20;41:307. doi: 10.1186/s13046-022-02518-8 (PMC9583503; doi:10.1186/s13046-022-02518-8)
Supplement: Supplementary file 9 — Additional file 9: Supplementary Table 3. Primers for qRT-PCR and ChIP. [file 13046_2022_2518_MOESM9_ESM.docx]

**Supplementary Table 3. Primers for qRT-PCR and ChIP**

| **Primer** | **Forward (5’-3’)** | **Reverse (5’-3’)** |
| --- | --- | --- |
| **qRT-PCR** | | |
| circLRFN5 | TCCCTGGAATACGTATGTTTCAAAT | CGGTGTCAAAAGATGGGTGC |
| PRRX2 | GCACCACGTTCAACAGCAG | TCCTTGGCCTTGAGACGGA |
| GCH1 | ACGAGCTGAACCTCCCTAAC | GAACCAAGTGATGCTCACACA |
| β-actin | CATGTACGTTGCTATCCAGGC | CTCCTTAATGTCACGCACGAT |
| **ChIP** | | |
| GCH1 | CGCCTAGAGCAGATGTATC | TCTCGAACTCCTGACCTC |
